# Nanoparticle-Mediated and Nanoengineered Drug Delivery for Glaucoma: Preclinical Evidence, Early Human Feasibility, and Translational Challenges

**DOI:** 10.3390/ph19101544

**Published:** 2026-09-29

**Authors:** Matteo Capobianco, Marco Zeppieri, Maria Letizia Salvetat, Francesco Pellegrini, Alessandra Pizzo, Irene Gattazzo, Fabiana D’Esposito, Francesco Cappellani, Caterina Gagliano

**Affiliations:** 1 Eye Clinic, Catania University, Policlinico Gaspare Rodolico, 95121 Catania, Italy; capobiancoteo@gmail.com; 2 Department of Ophthalmology, University Hospital of Udine, 33100 Udine, Italy; 3 Department of Medicine, Surgery and Health Sciences, University of Trieste, 34129 Trieste, Italy; 4 Department of Ophthalmology, Azienda Sanitaria Friuli Occidentale, Via Montereale 24, 33170 Pordenone, Italy; 5 Unità Operativa Complessa di Oftalmologia, Azienda Ospedaliera per l’Emergenza “Cannizzaro”, 95026 Catania, Italy; 6 Department of Ophthalmology, Ospedale Sant’Antonio, Azienda Ospedaliera, 35127 Padova, Italy; 7 Ophthalmic Research Group (ICORG) Unit, Imperial College, 153–173 Marylebone Rd, London NW1 5QH, UK; 8 Department of Medicine and Surgery, University of Enna “Kore”, Piazza dell’Università, 94100 Enna, Italy; 9 Mediterranean Foundation “Giovanni Battista Morgagni”, 95125 Catania, Italy

**Keywords:** glaucoma, intraocular pressure, nanoparticles, ocular hypertension, ocular surface

## Abstract

The management of glaucoma with drugs is mostly via the topical route, although these treatments have poor ocular bioavailability. Other problems limiting their therapeutic effect include pre-corneal clearance, corneal and ocular surface barriers, and the need for a long-term treatment regimen. Nanoparticle-mediated drug-delivery systems have shown the potential, particularly in preclinical studies, to improve ocular penetration and precorneal retention and to facilitate controlled drug release, with the aim of reducing dosing frequency. In this narrative review, the researchers focus on various nanoparticle-based approaches for sustained intraocular pressure control in glaucoma. The review encompasses carrier-based nanosystems, drug nanocrystals and nanosuspensions, nanoscale permeability or surface modifiers, multiscale nano-enabled formulations, and nanoparticle- or nanocarrier-integrated delivery devices. These approaches are designed to deliver conventional and innovative ocular hypotensive agents. Pharmacokinetics, corneal permeability, drug stability, tolerability, and duration of action of intraocular pressure-lowering drugs are critically reviewed across a predominantly preclinical evidence base; the two small human studies identified (*n* = 6 and *n* = 17) support clinical feasibility but do not establish durable clinical effectiveness, long-term safety, adherence benefit, or prevention of glaucoma progression. This review distinguishes improvements in ocular drug disposition from demonstrated IOP-lowering benefit and separates preclinical proof of concept from human evidence by applying an evidence-stratified formulation–performance framework rather than ranking nanocarriers by material class or isolated physicochemical properties. The available evidence is dominated by drugs that lower IOP, which in turn distinguishes prolonged control of IOP from any direct disease-modifying or neuroprotective action, for which glaucoma-specific evidence remains substantially more limited. The review further assesses the obstacles to clinical translation, including long-term safety, manufacturing reproducibility, sterilization, regulatory requirements, and patient and treatment burden. Generally, nanoengineered systems are promising platforms to address pharmacokinetic and treatment-delivery limitations of conventional glaucoma therapy, but comparative clinical effectiveness, long-term safety, adherence benefit, cost-effectiveness, and meaningful effects on glaucoma progression remain insufficiently demonstrated.

## 1. Introduction

Glaucoma comprises a heterogeneous group of progressive optic neuropathies characterized by retinal ganglion cell loss and optic nerve damage and remains a leading cause of irreversible blindness worldwide [1,2,3]. Its global burden is substantial and is expected to increase further with population ageing. A meta-analysis of 50 population-based studies estimated a glaucoma prevalence of 3.54% (95% credible interval [CrI], 2.09–5.82) among individuals aged 40–80 years, corresponding to 64.3 million affected people in 2013 and a projected 111.8 million by 2040 [1]. Despite considerable heterogeneity among glaucoma phenotypes, progressive retinal ganglion cell and axonal loss, thinning of the retinal nerve fiber layer, optic nerve head damage, and corresponding visual field loss represent common features of the disease. Intraocular pressure (IOP) is not invariably elevated—almost half of patients with glaucoma may have IOP within the statistically normal range—but it remains the only modifiable risk factor for which a therapeutic effect has been demonstrated in large clinical trials [2,3]. Accordingly, current glaucoma management is centered on lowering IOP toward an individualized target through medical, laser, or surgical treatment [2,3,4]. The clinical relevance of this strategy is supported across the continuum from ocular hypertension to established glaucoma. In the Ocular Hypertension Treatment Study, 1636 participants were randomized to topical hypotensive treatment or observation: after 60 months, the cumulative probability of developing primary open-angle glaucoma was 4.4% with treatment versus 9.5% with observation (hazard ratio [HR], 0.40; 95% CI, 0.27–0.59; *p* < 0.0001) [5]. Consistently, in 516 patients with newly diagnosed open-angle glaucoma enrolled in the United Kingdom Glaucoma Treatment Study, latanoprost reduced IOP by 3.8 mmHg at 24 months compared with 0.9 mmHg with placebo and significantly prolonged visual field preservation (adjusted HR, 0.44; 95% CI, 0.28–0.69; *p* = 0.0003) [6]. These findings establish IOP reduction as the central modifiable therapeutic strategy for preventing the onset or slowing the progression of glaucomatous damage. Various components of glaucoma pathophysiology must be distinguished conceptually. Elevated IOP represents an important modifiable risk factor and biomechanical stressor but does not equal glaucomatous neurodegeneration. Retinal ganglion cell degeneration represents progressive dysfunction and loss of the retinal output neurons, while damage to the optic nerve reflects injury and degeneration of their axons and associated optic nerve structures. Neuroinflammation, oxidative stress, mitochondrial dysfunction, and excitotoxicity are interacting cellular and molecular processes that are potential contributors to RGC and axonal injury, not simply alternative descriptors for either elevated IOP or optic-nerve damage [2,3]. Consequently, IOP reduction and direct neuroprotection deal with related but biologically different aspects of glaucoma.

The long-term effectiveness of topical therapy, however, depends on sustained and correct medication use, which remains a major limitation in glaucoma care. Non-adherence is multifactorial rather than attributable to a single behavior. In a cross-sectional survey of patients receiving glaucoma medications, 27% reported poor adherence and 61% identified multiple barriers; forgetfulness (OR, 5.6; 95% CI, 2.6–12.1), low self-efficacy (OR, 4.7; 95% CI, 2.2–9.7), difficulties with the medication schedule (OR, 2.9; 95% CI, 1.4–6.0), and difficulty instilling drops (OR, 2.3; 95% CI, 1.1–4.9) were independently associated with non-adherence [7]. This heterogeneity helps explain why isolated adherence interventions are unlikely to be universally effective. In a network meta-analysis of 19 randomized controlled trials including 4981 participants, tailored care improved adherence compared with standard care (standardized mean difference [SMD], 1.28; 95% CI, 0.08–2.48), with greater effects when combined with multimedia education (SMD, 1.44; 95% CI, 0.20–2.67) and a device reminder (SMD, 1.61; 95% CI, 0.75–2.47) [8]. The problem also extends to treatment persistence, with real-world evidence indicating substantial discontinuation during the first year of therapy [9].

Beyond adherence, prolonged topical exposure can compromise treatment tolerability through cumulative ocular surface toxicity. Ocular surface disease has been reported in 49–59% of patients receiving topical anti-glaucomatous medications, compared with approximately 5–30% in the general population older than 50 years, and the burden becomes particularly relevant because approximately 50–75% of patients may require multidrug therapy to achieve target IOP. Both active compounds and preservatives may contribute, but benzalkonium chloride (BAK) has been consistently associated with tear-film instability, goblet-cell loss, conjunctival inflammation and squamous metaplasia, epithelial apoptosis, and disruption of the corneal epithelial barrier, with toxicity that is both dose- and time-dependent. These ocular surface changes are clinically relevant because they may reduce treatment comfort, adherence, and quality of life, thereby adding a biological source of treatment burden to the behavioral limitations of chronic topical therapy [10,11].

Ocular drug delivery is hampered by a series of anatomical and dynamic impediments, the importance of which varies according to the route of administration and target tissue. The tear film is the first barrier to topical delivery at the ocular surface. The drug, which is instilled, is rapidly diluted and removed by blinking, tear turnover, reflex lacrimation, and nasolacrimal drainage, leaving only a short time for absorption. The conjunctiva is another potential route owing to its size and vascularity, which can permit drug absorption. However, because of conjunctival blood flow and lymphatic drainage, the drug may be removed from the ocular surface and may result in systemic exposure instead of intraocular exposure. As a result, only a small fraction of a conventional topical dose reaches intraocular tissues [12,13,14].

The corneal epithelium is the principal static barrier for drugs entering predominantly by the corneal route. Its lipophilic cellular layers and intercellular tight junctions restrict hydrophilic molecules and paracellular transport. On the other hand, the highly hydrated stroma restricts the diffusion of lipophilic compounds. After a drug reaches the anterior chamber, ocular distribution is influenced additionally by the blood–aqueous barrier, which is principally formed by the tight junction-containing non-pigmented ciliary epithelium and the vascular endothelium of the iris. It controls exchange between the systemic circulation and the aqueous compartment, and it is important for drugs delivered systemically or via routes that rely on access from periocular or vascular compartments [12,13,14].

The posterior segment of the eye presents different barriers than the anterior one. The sclera may offer a transscleral route from the conjunctival or periocular area, though diffusion through this tissue and subsequent access to deeper ocular structures depend on the physicochemical characteristics of the therapeutic system. The choroid is a highly vascularized structure that lies beyond the sclera. It can serve as an important clearance compartment, thus reducing the amount of drug that reaches the retina. In the case of intravitreal delivery, the barriers of the ocular surface and anterior segment are bypassed; however, the vitreous becomes the compartment for distribution, where diffusion, retention, and clearance govern access to retinal tissues. The neural retina has a blood–retinal barrier that limits exchange of substances between the systemic circulation and the neural retina. This barrier has two components: the inner barrier formed by tight junctions between retinal vascular endothelial cells and the outer barrier formed by the retinal pigment epithelium. The outer barrier separates the neural retina from the choroidal circulation [12,13].

Hence, these barriers should not be viewed as a single barrier to ocular delivery. Topical IOP-lowering therapy is dominated by tear-film clearance, conjunctival loss, and corneal epithelial penetration; periocular delivery must additionally contend with scleral transport and choroidal clearance. Injecting the formulation directly into the anterior chamber overcomes the obstacles presented by precorneal and corneal entry routes. On the other hand, intravitreal administration circumvents ocular-surface and anterior-segment entry barriers but introduces challenges related to vitreous distribution and retinal access. This route-dependent barrier profile is especially relevant for nanoengineered strategies that aim to shift focus from managing anterior-segment IOP toward retinal ganglion cell targeting or neuroprotection.

These pharmacokinetic constraints provide the rationale for developing ocular delivery systems capable of increasing residence time at the ocular surface while improving drug penetration, stability, solubility, and duration of action [14,15]. Nanotechnology-based carriers are particularly suited to this approach because their size, surface properties, composition, drug-loading characteristics, and release mechanisms can be modified to influence ocular retention and drug disposition. Platforms investigated for ocular delivery include lipid- and polymer-based nanoparticles, liposomes, solid lipid nanoparticles, nanostructured lipid carriers, niosomes, nanomicelles, nanoemulsions, and nanosuspensions, among other nanoscale systems [15,16]. Their potential in glaucoma is supported principally by preclinical evidence. A meta-analysis of 16 proof-of-concept studies evaluating topical particulate systems for IOP-lowering drugs in animal models found a significant increase in the area under the pharmacodynamic effect curve compared with conventional drug solutions (pooled estimate, 7.987; 95% CI, 5.334–10.639; *p* < 0.001). However, heterogeneity was very high (*I*^2^ = 91%), reflecting substantial differences among experimental models, measurements, study conditions, and sample sizes. Nanoparticles performed better than micron-sized particulate systems, whereas no significant difference emerged between lipid- and non-lipid-based carriers [17]. Thus, the available evidence establishes a strong experimental proof of concept for particulate ocular delivery, but its heterogeneity and exclusively animal basis in this meta-analysis preclude direct extrapolation to long-term clinical glaucoma management.

The literature therefore shifts the key question from whether nanocarriers can improve ocular drug disposition to whether these effects can be translated into reproducible and clinically acceptable formulations. An early analysis showed that sustained exposure requires both adequate ocular residence and an appropriate drug-release rate: excessively rapid release may negate prolonged exposure, whereas overly slow release may fail to generate the concentration gradient required for tissue penetration; at that time, reported ophthalmic nanoparticle studies remained confined to preclinical settings [18]. A subsequent glaucoma-focused review identified additional unresolved issues, including mechanisms of cellular entry, nanoparticle cytotoxicity, and demonstration of clinical efficacy [19]. Later analyses expanded these translational requirements to include physicochemical stability, biocompatibility, appropriate sterilization, batch-to-batch reproducibility, scalable manufacturing, quality control, and regulatory acceptability [20,21,22,23]. Importantly, the clinical development of sustained-release glaucoma therapy should not be conflated with that of nanoparticle-mediated delivery. Conventional sustained-release inserts, implants, and other delivery devices have reached human clinical development and, for selected technologies, clinical use [24], but this broader translational progress does not establish the clinical maturity of nanoparticle systems. Among the nanoparticle-specific platforms included in this review, human testing is limited to subconjunctival liposomal latanoprost and topical γ-cyclodextrin/dorzolamide nanoparticles, both evaluated in small early clinical studies. Accordingly, the nanoparticle-specific evidence base remains predominantly preclinical, and favorable in vitro or animal performance should be regarded as proof of concept rather than evidence of established clinical superiority.

The present narrative review critically examines nanoparticle-mediated drug-delivery strategies for glaucoma, with a particular focus on lipid-based nanoparticles, polymeric nanoparticles, nanomicelles, nanosuspensions and nanocrystals, and hybrid nanocarriers. The scope also includes larger or device-integrated delivery platforms only when a nanoscale component makes a direct functional contribution to drug loading, dissolution, retention, penetration, release, or ocular exposure; such systems are considered nano-enabled delivery architectures rather than conventional nanoparticle formulations. Beyond this platform-based description, the review applies an evidence-stratified formulation–performance framework centered on four clinically relevant questions: which formulation characteristics improve ocular retention, penetration, or drug exposure; which of these improvements translate into greater or more durable IOP control; whether these formulation–performance relationships are reproducible across drugs, experimental models, administration routes, and study designs; and which delivery strategies have generated credible human evidence. Physicochemical or pharmacokinetic improvement is therefore not considered a surrogate for therapeutic superiority, and preclinical proof of concept is explicitly distinguished from human evidence. Attention is given to their effects on precorneal residence, corneal permeability, pharmacokinetics, duration of IOP reduction, ocular tolerability, and the possibility of reducing administration frequency. Available human evidence is distinguished from preclinical findings, and the principal challenges related to toxicity, formulation stability, manufacturing scalability, reproducibility, sterilization, regulatory characterization, and clinical translation are discussed.

**Figure 1:**
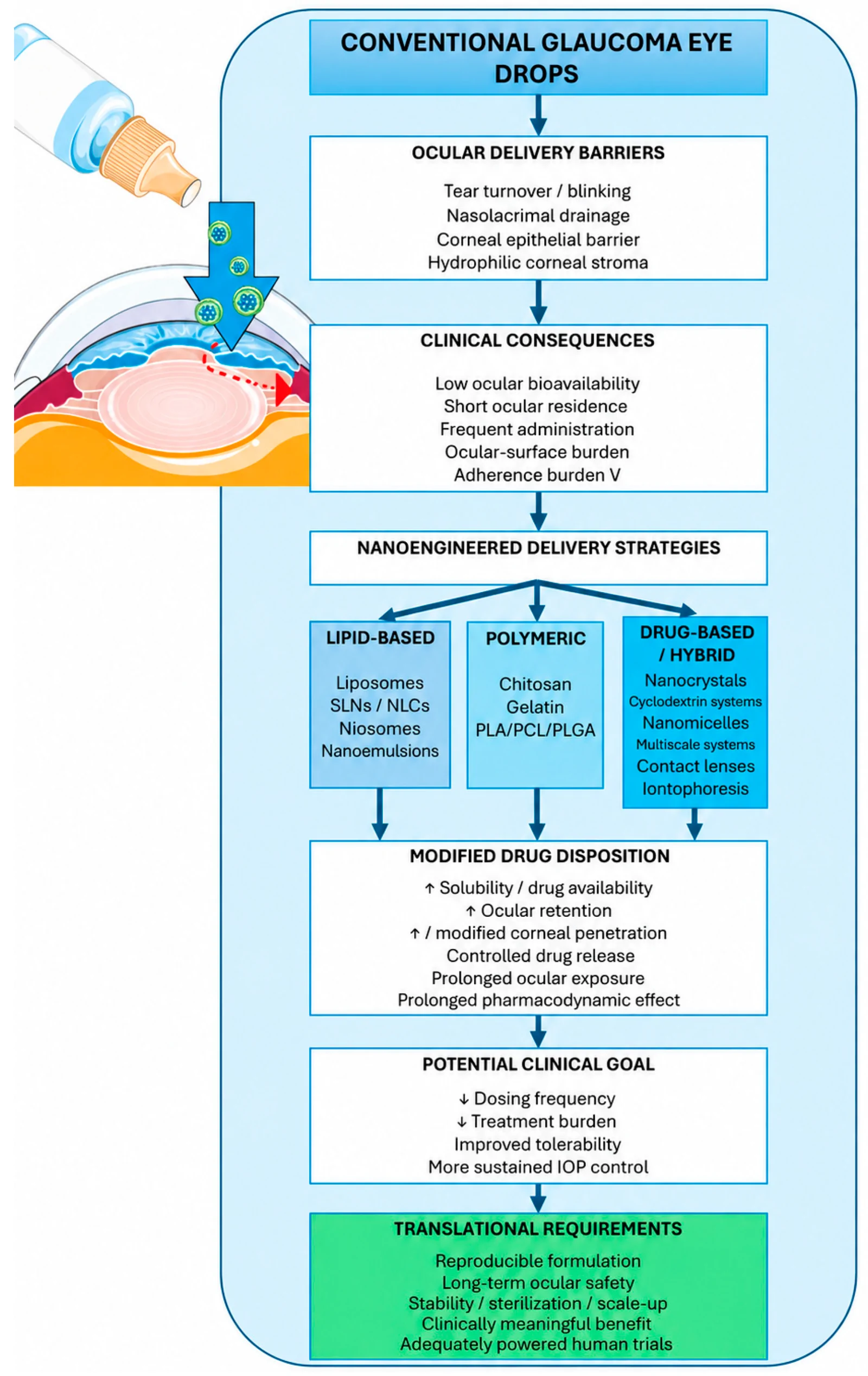
Schematic representation of the rationale for nanoengineered drug delivery in glaucoma. Precorneal clearance and corneal barriers limit conventional topical drug exposure and contribute to frequent administration and treatment burden. Nanoengineered platforms can modify drug solubility, ocular retention, penetration, and release to prolong pharmacological exposure. Translation of these effects into clinical benefit requires reproducible formulation, long-term ocular safety, scalable manufacturing, and confirmation in adequately powered human studies. Image(s) provided by Servier Medical Art (https://smart.servier.com, accessed on 28 August 2026), licensed under CC BY 4.0 (https://creativecommons.org/licenses/by/4.0/, accessed on 28 August 2026).

The main limitations of conventional topical glaucoma therapy and the rationale for nanoengineered drug-delivery systems are summarized in Figure 1.

## 2. Results

The findings of each individual study are presented below by delivery platform, along with evidence detailing their formulation-level findings. The cross-platform interpretation of findings is organized in Section 3 under ocular exposure, pharmacodynamic performance, reproducibility, and level of clinical evidence. Quantitative outcomes are reported using the definitions, units, denominators, comparator conditions, and time intervals specified in the individual primary studies. As such, reported fold changes, relative bioavailability estimates, permeability ratios, AUC values, residence-time ratios, and percentage IOP reductions are descriptive measures specific to each study and should not be regarded as standardized or directly comparable effect sizes across studies.

### 2.1. Nanocarrier Design and Lipid-Based Systems

Early experimental work illustrates that the performance of ocular nanocarriers depends not only on the material class but also on structural features that govern drug incorporation, penetration, and release. Attama et al. formulated timolol hydrogen maleate in phospholipid surface-modified solid lipid nanoparticles (SM-SLNs) and evaluated their transport across a bioengineered human corneal construct. Surface modification produced particles of 37.7–47.2 nm, compared with >160 nm for unmodified SLNs, with encapsulation efficiencies > 44% and sustained in vitro release. Importantly, the free timolol solution showed a higher permeation coefficient (4.27 × 10^−5^ cm/s) than the SM-SLN formulations (1.33–2.31 × 10^−5^ cm/s), whereas the latter provided more sustained permeation without a burst-release pattern [25]. Thus, the principal advantage demonstrated was control of the release and permeation profile rather than an increase in instantaneous transcorneal flux; no in vivo efficacy experiment was performed.

The importance of nanocarrier architecture was demonstrated more directly by Nguyen et al. using pilocarpine-loaded hollow poly(lactic acid) nanoparticles with shell thicknesses ranging from approximately 10 to 100 nm. Increasing shell thickness improved sustained release but progressively reduced drug incorporation: encapsulation efficiencies declined from 93 ± 0.2% with ~10-nm shells to 52 ± 0.3% with ~100-nm shells, whereas the ~40-nm formulation provided an intermediate encapsulation efficiency of 86 ± 0.3% and maintained pilocarpine concentrations above the therapeutic level of approximately 10 μg/mL for more than 56 days. A single intracameral administration of this ~40-nm-shell formulation sustained control of ocular hypertension for more than 56 days in experimentally glaucomatous rabbits and was associated with preservation of corneal endothelial, retinal, and optic-nerve structure and function [26]. These findings demonstrate that prolonged exposure is not simply a function of maximizing drug loading but may require optimization between loading capacity and release kinetics.

Luo et al. extended this structure–function concept by combining delivery and intrinsic carrier activity within a dual-functional hollow ceria nanoparticle platform. Chitosan and the adenosine A2A receptor antagonist ZM241385 were functionalized onto hollow ceria nanoparticles loaded with pilocarpine, with the formulation designed to facilitate corneal epithelial tight-junction opening and preferential delivery toward the ciliary body. In experimentally glaucomatous eyes, a single topical administration maintained therapeutic activity for 7 days, compared with approximately 4 h for conventional pilocarpine eye drops, and produced an approximately 250-fold increase in pilocarpine bioavailability in the ciliary body [27]. The ceria component also displayed antioxidant and anti-inflammatory activity in the experimental assessments, illustrating a broader strategy in which the nanocarrier may contribute biologically in addition to controlling drug disposition.

Among the platforms considered here, liposomal latanoprost provides the most direct evidence of clinical translation. Wong et al. conducted a first-in-human, open-label, non-comparative pilot study in six subjects—five with ocular hypertension and one with primary open-angle glaucoma—who received a single subconjunctival injection of liposomal latanoprost. Mean IOP decreased from 27.55 ± 3.25 mmHg to 14.52 ± 3.31 mmHg within 1 h, corresponding to a mean reduction of 13.03 ± 2.88 mmHg or 47.43 ± 10.05%. A reduction of at least 20% remained statistically significant through 3 months (*p* = 0.001–0.049). All six subjects completed follow-up without loss of visual acuity; transient discomfort was common immediately after injection, while one patient with pre-existing ocular surface disease reported discomfort and intermittent blurred vision that improved with lubricants [28]. These findings provide human proof of concept for prolonged nanoliposomal IOP lowering, but the sample size, absence of masking and comparator, and non-randomized design preclude conclusions regarding comparative efficacy or long-term safety.

Alongside liposomal systems, amphiphilic nanomicelles represent a distinct approach in which self-assembled structures can enhance the solubilization and ocular delivery of poorly soluble compounds. Recent analysis of ophthalmic nanomicelles emphasizes their tunable core–shell architecture, small dimensions, surface modification, and ability to alter drug retention and epithelial permeation; however, most applications remain at the preclinical or early translational stage. In glaucoma specifically, the available literature includes nanomicelle-loaded contact-lens systems for combined timolol and latanoprost delivery in rabbits, illustrating the possibility of integrating nanocarriers into larger sustained-delivery devices rather than using them exclusively as conventional eye-drop dispersions [29].

More recent liposomal development has increasingly combined prolonged hypotensive activity with preservation of the ocular surface. Brugnera et al. formulated 0.005% latanoprost in synthetic phosphatidylcholine liposomes dispersed in 0.2% hyaluronic acid and supplemented with the osmoprotectants betaine (0.40%) and leucine (0.90%). The optimized LAT-HA-LIP formulation had a mean vesicle size of 195.14 ± 14.34 nm and a zeta potential of −13.96 ± 0.78 mV. The measured total latanoprost concentration was 52.26 ± 2.05 μg/mL, corresponding to 104.52 ± 4.10% of the nominal target concentration, while free latanoprost was below the analytical limit of detection and encapsulation efficiency was ≥99.46%. In rabbits, a single topical dose maintained its hypotensive effect for 48 h compared with 24 h for the marketed formulation. Based on the area under the IOP-effect curve, the authors reported a 2.97-fold greater relative ocular bioavailability than the reference formulation (*p* = 0.0006). Cell viability exceeded 80% in corneal and conjunctival epithelial models, and short-term rabbit testing showed no significant ocular-surface damage or discomfort, although minor conjunctival hyperemia occurred with both LAT-HA-LIP and the latanoprost comparator [30].

The subsequent study of the same preservative-free platform addressed repeated exposure, tissue penetration, and ocular-surface parameters. In an ex vivo porcine-eye model, the liposomal formulation increased penetration of latanoprost acid across corneal and conjunctival–scleral tissues compared with the marketed benchmark, with latanoprost acid detected beyond the conjunctiva, sclera, and choroid only after LAT-HA-LIP administration. During rabbit tolerance studies, Schirmer testing, tear osmolarity, tear breakup time, and fluorescein staining remained broadly within physiological ranges over a 25-day monitoring period; on day 15, tear breakup time was 7.00 ± 0.78 s with LAT-HA-LIP versus 6.00 ± 0.63 s with saline control (*p* = 0.0066). Daily administration for 15 consecutive days maintained mean IOP reductions of 2.51–3.88 mmHg between days 5 and 15 [31]. Nevertheless, the authors themselves noted that efficacy was assessed over only 15 days and that studies extending beyond 6 months or longer would be required to establish chronic safety and efficacy.

Taken together, these studies show that nanocarrier performance is determined by a balance among carrier composition, architecture, drug loading, tissue interaction, and release kinetics rather than by nanoscale dimensions alone. Liposomal systems are particularly notable because they have progressed from controlled in vitro delivery to topical animal formulations and, in the case of subconjunctival latanoprost, preliminary human evaluation. However, the marked differences in carrier composition, administration route, experimental model, and duration of follow-up prevent direct comparison of efficacy across platforms.

Lipid-based formulations have also been investigated for the delivery of the α2-adrenergic agonist brimonidine, with carrier architecture affecting drug incorporation and pharmacodynamic duration. El-Salamouni et al. directly compared brimonidine-loaded nanostructured lipid carriers (NLCs) with solid lipid nanoparticles (SLNs) and commercial eye drops. The optimized NLCs measured 151.97 ± 1.98 nm, had an entrapment efficiency of 83.631 ± 0.495%, and remained stable for 3 months at 4 °C. Their permeability coefficient was 1.227-fold higher than that of SLNs, although this difference was not statistically significant (*p* > 0.05), and both lipid formulations showed lower total corneal permeation than free brimonidine preparations. Nevertheless, histological examination demonstrated anterior-segment localization, and the in vivo pharmacodynamic profile was prolonged: NLCs produced an IOP reduction of −4.27 ± 0.76 mmHg after 0.5 h and a maximum decrease of −13.14 ± 1.28 mmHg, compared with −0.95 ± 0.07 and −10.03 ± 0.32 mmHg, respectively, for SLNs; both nanoparticle formulations maintained IOP-lowering activity for >24 h [32].

A different vesicular strategy was evaluated by Eldeeb et al., who used a proniosomal gel as a precursor from which brimonidine-loaded niosomes were generated after hydration, specifically addressing aggregation, fusion, and drug leakage associated with conventional niosomal dispersions. The optimized Span 60-based formulation showed an entrapment efficiency of 79.23 ± 1.12%, a particle size of 810.95 ± 16.76 nm, and cumulative drug release of 40.98 ± 1.29% at 2 h, 63.35 ± 6.07% at 8 h, and 91.11 ± 1.76% at 24 h. In rabbits, maximum IOP reduction was 41.32 ± 2.42% at 2 h, compared with 34.49 ± 3.60% for Alphagan^®^ P; the commercial formulation returned to baseline after 5 h, whereas the proniosomal formulation retained an IOP-lowering effect at 24 h (6.53 ± 2.92%). Its pharmacodynamic AUC0–24, calculated from the percentage IOP-decrease–time curve, was 5.024-fold that of Alphagan^®^ P, and the corresponding pharmacodynamic MRT was increased 7.90-fold [33].

The same principle of modifying lipid composition to alter ocular drug disposition has been applied to carbonic anhydrase inhibitors. Jin et al. incorporated brinzolamide into D-α-tocopheryl polyethylene glycol 1000 succinate (TPGS)-modified nanoliposomes with a mean diameter of 96.87 ± 4.43 nm, an entrapment efficiency of 95.41 ± 3.03%, and drug loading of 4.00 ± 0.13%. The apparent transcorneal permeability coefficient was 10.2-fold higher than that of commercial AZOPT^®^ and 1.38-fold higher than that of unmodified brinzolamide liposomes. The formulation also prolonged precorneal residence, with higher tear-fluid brinzolamide concentrations than the comparator formulations at later time points. Correspondingly, effective IOP reduction persisted from 3 to 11 h with TPGS-modified liposomes, compared with 3–6 h for AZOPT^®^ and 3–8 h for conventional liposomes [34]. Preliminary irritation and histological assessments did not identify significant corneal injury.

Lipid carriers can also accommodate more than one hypotensive agent. Shrivastava et al. co-loaded timolol maleate and brinzolamide into an NLC formulation with a particle size of 110.36 ± 0.47 nm and a polydispersity index of 0.24 ± 0.00. Entrapment efficiencies were 77.12 ± 0.64% for timolol and 70.73 ± 0.64% for brinzolamide. Approximately 34 ± 2.90% of timolol and 38 ± 3.10% of brinzolamide were released during the first 5 h, followed by cumulative release of 72.29 ± 5.90% and 70.08 ± 6.40%, respectively, at 24 h. Ex vivo penetration followed a similarly extended profile, reaching 72.30 ± 6.40% for timolol and 67.69 ± 6.50% for brinzolamide at 24 h and exceeding that obtained from the corresponding drug suspensions [35]. Because the study was limited to physicochemical, in vitro, and ex vivo evaluation, these findings demonstrate feasibility of dual-drug incorporation and delivery but not IOP-lowering efficacy.

For the highly lipophilic prostaglandin analogue travoprost, both nanoemulsions and liquid crystalline nanostructures have been used to increase ocular exposure. In the formulation developed by Ismail et al., the selected nanoemulsion contained droplets of approximately 157 nm and remained physically stable after filtration and accelerated storage. In rabbits, travoprost reached the same Tmax of 8 h with the nanoemulsion and Travatan^®^, but Cmax increased from 0.57 to 1.205 µg/mL and AUC0–∞ from 4.17 to 8.032 µg·h/mL (*p* < 0.05 for both comparisons). The nanoemulsion maintained normal IOP through 60 h, whereas Travatan^®^ did so through 36 h; both preparations were classified as non-irritant in the ocular tolerability assessment [36].

El-Gendy et al. subsequently evaluated travoprost-loaded liquid crystalline nanostructures composed of glyceryl monooleate with different penetration enhancers. Two selected formulations, F-1-L and F-3-L, measured 216.20 ± 6.12 and 129.40 ± 11.73 nm and achieved entrapment efficiencies of 85.30 ± 4.29% and 82.54 ± 7.65%, respectively. F-3-L produced the highest 8-h ex vivo corneal permeation among the selected formulations (144.07 ± 1.60 µg/cm^2^), compared with 21.59 ± 7.17 µg/cm^2^ for the drug solution. In rabbits, relative ocular bioavailability was 106.1% for F-1-L and 322.82% for F-3-L compared with Travatan^®^, while IOP-lowering activity persisted for 48 and 72 h, respectively, versus 36 h with the marketed formulation. F-3-L also increased aqueous-humor Cmax to 1.80 ± 0.15 ng/mL versus 1.42 ± 0.09 ng/mL with Travatan^®^ and prolonged median Tmax from 1 to 6 h; no ocular injury was detected in the reported tolerability evaluation [37].

Taken together, these lipid-based studies show that corneal permeability, tissue exposure, residence time, release kinetics, and pharmacodynamic duration are related but non-equivalent outcomes. Surface-modified timolol SLNs prolonged permeation despite a lower permeability coefficient than free timolol [25], whereas brimonidine-loaded NLCs and SLNs showed lower total corneal permeation than free brimonidine preparations while still prolonging IOP-lowering activity beyond 24 h [32]. By contrast, TPGS modification of brinzolamide liposomes increased apparent transcorneal permeability by 10.2-fold relative to AZOPT^®^ and was accompanied by prolongation of effective IOP reduction from 3–6 h to 3–11 h [34]. These differences indicate that prolonged pharmacodynamic activity may arise through different formulation mechanisms and should not be attributed automatically to greater corneal permeability. The relevant causal interpretation therefore depends on the complete concentration–time profile generated by the formulation, including release, ocular residence, penetration, tissue exposure, and drug availability at the target site. The evidence in this group nevertheless remains predominantly preclinical, and improvements in any single pharmacokinetic or formulation endpoint should not be interpreted as sufficient evidence of superior therapeutic performance. The boundaries between conventional lipid carriers and more engineered hybrid systems are not absolute, as illustrated by the lipid–DNA nanoparticles developed by Schnichels et al. In this platform, 12-nucleotide amphiphilic DNA strands self-assembled into particles approximately 10 nm in diameter and travoprost was incorporated through a specific aptamer with a dissociation constant of 104 nM, allowing non-covalent drug loading without chemical modification of the active compound. In rats, fluorescently labelled nanoparticles remained detectable on the corneal epithelium for up to 60 min after topical administration, whereas the free labelled aptamer was no longer detectable after 15 min. Quantitative LC–MS analysis in porcine corneas confirmed that this prolonged surface association increased local drug delivery: immediately after exposure, travoprost concentrations were 1.77 ng/mg with the nanoparticle formulation versus 0.60 ng/mg with free travoprost, and remained higher after 5, 30, and 60 min of washing (0.95 vs. 0.50, 0.88 vs. 0.30, and 1.16 vs. 0.48 ng/mg, respectively; *p* < 0.001 at each time point). In vivo LC–MS measurements likewise showed at least a twofold increase in drug delivery at the investigated time points and approximately fourfold greater delivery 4 h after instillation. Importantly, the pharmacodynamic findings were less definitive: 12 h after treatment, IOP decreased in 10/20 eyes (50%) receiving travoprost-loaded nanoparticles and 6/18 eyes (33%) receiving free travoprost; among responding eyes, mean relative IOP reductions were 30 ± 16% and 17 ± 11%, respectively, but the difference was not statistically significant (*p* = 0.1) [38]. Thus, this study provides strong evidence for improved residence and drug delivery, while superiority in IOP lowering remains unconfirmed.

The importance of formulation architecture is also evident in niosomal delivery of acetazolamide. Guinedi et al. compared reverse-phase evaporation (REV) and multilamellar vesicles (MLVs) containing Span 40 or Span 60 with different cholesterol ratios. Entrapment depended on all three variables—surfactant, cholesterol content, and preparation method—and was highest with Span 60 MLVs at a 7:6 surfactant-to-cholesterol molar ratio (32.21 ± 0.34%); however, this formulation was not the most effective pharmacodynamically. Span 60 formulations at a 7:4 ratio provided greater drug release over 8 h, and the corresponding MLV formulation produced the most prolonged hypotensive response. After a single topical dose in normotensive rabbits, free 1% acetazolamide produced a maximum IOP reduction of no more than 3.7 mmHg at 1 h, whereas 7:4 Span 60 REV and MLV niosomes produced reductions of 7.15 and 6.90 mmHg at 1 h and 6.10 and 7.75 mmHg at 4 h, respectively; the effect of the niosomal formulations persisted throughout the 8-h observation period (*p* < 0.01 versus free acetazolamide and plain niosomes). The optimized MLV formulation retained 77.12% of encapsulated acetazolamide after 3 months under refrigerated storage. After once-daily administration for 40 days, histology showed only slight stromal edema, described as reversible, without major corneal tissue changes [39]. These findings reinforce that maximizing entrapment alone does not necessarily maximize ocular pharmacodynamic performance, because vesicle composition and lamellarity simultaneously influence drug retention and release.

Taken together with the preceding studies, these data show that lipid-containing and vesicular nanocarriers can be engineered at multiple structural levels—from lipid composition and lamellarity to DNA-based molecular recognition—to regulate drug loading, ocular residence, and release. However, the evidence reviewed in this subsection remains overwhelmingly preclinical; among the systems discussed, the previously described six-patient liposomal-latanoprost study remains the principal human proof of concept rather than evidence of established clinical efficacy.

### 2.2. Polymeric Nanoparticles

Polymeric nanoparticles provide a complementary approach to lipid-based systems because drug release and ocular disposition can be modified through polymer composition, surface properties, and the formulation vehicle in which the particles are administered. Natural polymers have been investigated particularly for topical delivery. Using ionic gelation, Saroha et al. prepared timolol maleate-loaded chitosan nanoparticles and identified an optimized formulation with a particle size of 190.9 nm and an entrapment efficiency of 75.34 ± 0.17%; cumulative in vitro drug release reached 49.11 ± 0.49% after 12 h [40]. Because this study was limited to formulation development and in vitro release, it establishes controlled-release feasibility but does not provide evidence of enhanced corneal delivery or IOP lowering.

The subsequent gelatin-based work by Shokry et al. extended this concept to in vivo evaluation. Their optimized timolol-loaded gelatin nanoparticles measured approximately 205 nm, had a zeta potential of +12.5 mV and an entrapment efficiency of 74.72%, and showed an initial burst followed by sustained release. In rabbits with experimentally induced ocular hypertension, the selected 1:2 NH_2_: glutaraldehyde formulation produced a smoother 24-h IOP profile than marketed 0.5% timolol: mean IOP over the observation period was approximately 20 versus 28 mmHg, corresponding to average reductions from baseline of 52% and 31%, respectively. Depending on the tonometer plunger load, the lowest recorded IOP with this formulation was 18.13 ± 1.65 or 14.48 ± 2.46 mmHg, compared with 25.50 ± 1.27 or 25.15 ± 2.37 mmHg with marketed timolol, while the corresponding AUC values were also significantly greater (*p* < 0.05). The nanoparticle formulations produced no Draize-detectable irritation; histological evaluation after repeated administration showed mild-to-moderate edema during the first 2 weeks that subsequently regressed, with normal retinal and ciliary-body structures reported by the end of week 3 rather than an entirely unchanged histological profile throughout treatment [41].

More recent chitosan formulations have focused more directly on corneal transport. Alshweiat et al. prepared low-molecular-weight chitosan/timolol complexes measuring 185–258 nm, with positive zeta potentials >+26 mV and encapsulation efficiencies of 28–35%; incorporation into liquid medicated nanoparticle formulations reduced particle size to 65–130 nm. Timolol release was sustained for 24 h and followed the Korsmeyer–Peppas model with *n* < 0.45, consistent with Fickian diffusion. In excised sheep cornea, the best-performing formulation produced a flux of 2.54 ± 0.04 μg·cm^−2^·h^−1^ and a permeability coefficient of 0.00987 cm·h^−1^, corresponding to a 2.62-fold increase in timolol permeation [42]. However, no in vivo pharmacodynamic experiment was performed, so the observed gain concerns corneal permeation rather than demonstrated IOP-lowering efficacy.

Synthetic biodegradable polymers allow the contribution of the nanoparticle itself to be distinguished from that of the surrounding ophthalmic vehicle. Ibrahim et al. compared brimonidine-loaded PCL, PLGA, and PLA nanoparticles, with mean particle sizes of 117.33 ± 4.58, 125.67 ± 5.15, and 131.67 ± 3.79 nm and encapsulation efficiencies of 77.97 ± 1.38%, 68.65 ± 3.35%, and 73.52 ± 2.92%, respectively. The particles were incorporated into eye drops, a temperature-triggered in situ gel, or a preformed gel. Although the maximal IOP-lowering effect did not differ significantly from Alphagan^®^ P in either high- or normal-IOP mice (*p* > 0.05), the nanoparticle formulations significantly delayed the time to maximal response and extended the duration of IOP lowering; AUC was greater than with Alphagan^®^ P, with the overall pharmacodynamic ranking preformed gel > in situ gel > eye drops and, among the polymers, PCL > PLGA > PLA [43]. Thus, the study showed that sustained pharmacodynamic performance was determined by the combination of polymer and dosage-form vehicle rather than polymer selection alone.

This interaction between carrier and vehicle became even clearer when the same group replaced the synthetic polymers with the bioadhesive natural polymers chitosan and alginate. Brimonidine-loaded chitosan and alginate nanoparticles measured 115.67 ± 3.58 and 157.67 ± 5.53 nm and achieved encapsulation efficiencies of 74.34 ± 2.05% and 70.40 ± 2.77%, respectively. When incorporated into the same three types of topical vehicles, the formulations did not significantly increase the maximal IOP reduction relative to Alphagan^®^ P (*p* > 0.05), but shifted Tmax from 2.2–2.4 h with the marketed drops to 5.6–7.2 h and extended the duration of IOP lowering from 7.0–7.4 h to 17.2–25.2 h (*p* < 0.001). Cell viability remained between 88.78% and 97.43%, compared with 89.79% for Alphagan^®^ P. Notably, the authors directly contrasted these results with their earlier synthetic-polymer study, in which the effect lasted up to approximately 16 h, and attributed the further prolongation to the combined bioadhesive contribution of the natural-polymer nanoparticles and the formulation vehicle [44].

Surface modification provides another means of changing polymeric-carrier behavior without altering the encapsulated drug. Shahab et al. coated dorzolamide-loaded PCL nanoparticles with chitosan, obtaining particles of 192.38 ± 6.42 nm with a PDI of 0.18 ± 0.04, a zeta potential of +5.21 ± 1.24 mV, and an encapsulation efficiency of 72.48 ± 5.62%. Drug release was biphasic, with an initial 2-h burst followed by sustained release through 12 h. More importantly, ex vivo corneal permeation increased from 94.23 ± 4.12 μg/cm^2^ with dorzolamide solution to 195.23 μg/cm^2^ with the chitosan-coated nanoparticles (*p* < 0.05), while mucoadhesive strength increased from 18.11% to 67.54%, a 3.7-fold difference [45]. Corneal hydration remained within the reported physiological range at 76.45%, and both histopathology and HET-CAM testing supported ocular tolerability. Because no in vivo IOP experiment was included, these findings demonstrate improved mucoadhesion and corneal transport rather than pharmacodynamic superiority.

Polymeric carriers can also be used to bypass the precorneal compartment entirely. Salama et al. administered brinzolamide-loaded PLGA nanoparticles by a single subconjunctival injection in rabbits and showed that particle characteristics materially influenced subsequent drug exposure. The smaller selected formulation, A19 (193 ± 0.40 nm), produced a more prolonged IOP-lowering response than the larger B11 formulation (660.75 ± 51.61 nm), and their AUC0–10 days values were respectively 9.2- and 7.1-fold higher than that of brinzolamide suspension (*p* < 0.05). The nanoparticles reduced IOP for up to 10 days after a single injection, despite in vitro release extending for much longer periods, underscoring the absence of a direct in vitro–in vivo correlation in this model. Local tolerability was generally favorable but not completely devoid of tissue changes: the transient subconjunctival bleb and vascularization resolved within 3 days, whereas histology showed iris vascular congestion with A19 and mild reversible retinal degeneration with B11, without severe inflammation or alteration of the cornea, choroid, or scleral endothelium [46].

Taken together, these studies show that polymeric ocular delivery is governed by the complete dosage form rather than by nanoparticle composition alone. In the brimonidine studies, changes in polymer identity, bioadhesion, and external vehicle altered Tmax, exposure, and duration of IOP lowering without significantly increasing the maximal IOP reduction relative to Alphagan^®^ P [43,44]. The principal pharmacodynamic advantage was therefore prolongation of exposure and response rather than enhancement of peak hypotensive potency, and the contribution of the nanoparticle cannot be separated completely from that of the surrounding vehicle. Similarly, chitosan surface modification of dorzolamide-loaded PCL nanoparticles markedly increased mucoadhesion and ex vivo corneal permeation, but the absence of an in vivo IOP experiment limits this finding to mechanistic and transport evidence rather than therapeutic superiority [45]. The subconjunctival PLGA study further demonstrates that in vitro release duration is not a reliable surrogate for in vivo pharmacodynamic persistence: IOP lowering lasted approximately 10 days despite substantially longer in vitro release, while particle size affected exposure and mild reversible tissue changes were observed [46]. Thus, polymer structure, surface properties, external vehicle, particle size, administration route, and biological disposition collectively determine performance, and neither prolonged release nor enhanced ex vivo permeation alone establishes superior glaucoma treatment.

### 2.3. Nanocrystals, Cyclodextrin-Based Nanoparticles, and Multiscale Delivery Systems

Nanocrystal formulations differ fundamentally from carrier-based nanoparticles because the nanoscale dispersed phase consists predominantly of the active drug itself, while relatively small amounts of excipients stabilize the crystal surface. Tuomela et al. applied this approach to the poorly water-soluble carbonic anhydrase inhibitor brinzolamide using top-down wet milling. Among the stabilizers tested, poloxamers F68 and F127 and polysorbate 80 failed to generate stable nanocrystals, producing heterogeneous particles > 1000 nm, whereas hydroxypropyl methylcellulose (HPMC) yielded homogeneous suspensions. The final formulations had mean particle sizes of 460 ± 10 nm at pH 7.4 and 530 ± 2 nm at pH 4.5, with polydispersity indices of 0.21 ± 0.17 and 0.12 ± 0.02, respectively, and remained physically stable at 5 °C for 4 months [47].

Particle-size reduction markedly changed dissolution without altering the crystalline state of brinzolamide. Approximately 100% of the drug from all three nanocrystal formulations dissolved within 1 min in PBS at pH 7.4, whereas only approximately 50% of unmilled bulk brinzolamide had dissolved after 30 min; dissolution was similar to that of Azopt^®^. In HCE-T cells, undiluted formulations reduced viability after 5 min to 70%, 85%, and 77% for formulations I–III, compared with 69% for Azopt^®^, whereas physiologically more relevant 1:10 and 1:52 dilutions produced only mild or no toxicity. In laser-induced ocular hypertension in rats, all nanocrystal formulations reduced IOP after a single 100-μg topical dose. At 60 min, reductions from the elevated baseline were 55.9 ± 20.7%, 51.0 ± 26.3%, and 71.4 ± 5.0% for formulations I–III, respectively, compared with 49.6 ± 16.5% for Azopt^®^. The pH 4.5 formulation produced a significantly greater reduction than formulation II and Azopt^®^ at this time point, suggesting that formulation conditions influencing the dissolved/free-drug fraction remained relevant even when dissolution of the nanosized preparations was similarly rapid in vitro [47]. Thus, accelerated dissolution was an important consequence of nanosizing but did not, by itself, explain the differences in pharmacodynamic response among formulations. The divergence in IOP lowering despite similarly rapid in vitro dissolution indicates that formulation-dependent factors affecting the dissolved/free-drug fraction and subsequent ocular availability remained important determinants of biological performance.

Cyclodextrin-based particles use a different mechanism, combining drug solubilization with self-assembly and ocular-surface retention. Gudmundsdottir et al. evaluated aqueous 3% dorzolamide/γ-cyclodextrin nanoparticles in the first human clinical trial of this formulation, using a prospective randomized single-masked crossover design in 17 untreated subjects with IOP > 18 mmHg. A single daily nanoparticle dose was compared with Trusopt administered three times over 24 h. At 4 h, the peak IOP reduction was 3.8 ± 2.6 mmHg (18%) with the nanoparticle formulation and 3.1 ± 3.7 mmHg (14%) with Trusopt; both reductions were significant from baseline, but there was no difference between treatments (*p* = 0.97). At 24 h, reductions were 1.4 ± 2.8 mmHg (6%) and 1.5 ± 2.0 mmHg (7%), respectively, neither being significant from baseline and with no between-group difference (*p* = 0.23). Burning was substantially lower with the nanoparticle drops (visual analogue score 12 ± 15 vs. 37 ± 30; *p* = 0.0038), while visual acuity and conjunctival hyperemia did not differ between treatments [48]. Thus, under the specific conditions of this small, short-term study, one administration of the cyclodextrin formulation produced a broadly comparable 24-h IOP profile to three administrations of commercial dorzolamide, while substantially reducing burning scores. However, the absence of statistically significant between-treatment differences should not be interpreted as evidence of therapeutic equivalence or non-inferiority, which was not established by a prospectively defined equivalence or non-inferiority margin. The study therefore provides preliminary evidence of clinical feasibility and short-term tolerability rather than proof that one nanoparticle dose is therapeutically equivalent to three conventional doses. Park et al. extended the same objective of prolonging preocular exposure through a multiscale system rather than a conventional nanoparticle suspension. Dorzolamide-loaded PLGA/PEG nanofibers were electrospun, freeze-milled into mucoadhesive nanostructured microparticles (MUCO_NM), and incorporated into a rapidly dissolving PVA tablet. The resulting particles remained microscale (1.61 ± 0.17 μm) but had a specific surface area of 34.18 m^2^/g, compared with 3.22 m^2^/g for smooth mucoadhesive microspheres, while containing 134.7 ± 3.84 μg dorzolamide/mg. This architecture translated into a graded pharmacodynamic effect: Trusopt produced a maximum IOP decrease of 17.96 ± 1.62% at 2 h, lasting 5 h, with an AUC of 51.46 ± 7.74; the MUCO_NM formulation reached 24.40 ± 2.31% at 3 h, persisted for 10 h, and increased AUC to 115.85 ± 12.30. Intermediate spherical or non-mucoadhesive formulations produced smaller gains, supporting a combined contribution from surface nanostructure and mucoadhesive design rather than from the tablet vehicle alone. At the same dorzolamide dose, the MUCO_NM formulation therefore approximately doubled both duration and overall IOP-lowering exposure compared with Trusopt, and its pharmacodynamic profile was comparable to either two Trusopt administrations separated by 4 h or a single four-drop dose [49].

Together, these approaches show that nanoscale engineering does not require a single carrier paradigm. Reducing the drug itself to nanocrystals can primarily alter dissolution and the available drug fraction [47]; cyclodextrin self-assembly can combine solubilization with prolonged ocular-surface availability and has already reached initial human evaluation [48]; and nanostructuring can also be incorporated into larger mucoadhesive systems to increase surface interaction while retaining a microscale final dosage form [49]. The level of evidence remains heterogeneous, as the nanocrystal and multiscale mucoadhesive approaches have so far been evaluated only in preclinical models, whereas cyclodextrin-based dorzolamide has reached initial human testing in a small, short-term crossover trial [47,48,49].

### 2.4. Nanomicelles, Hybrid Systems, and Combination Approaches

The versatility of nanoparticulate design is also evident in formulations of pilocarpine, in which physicochemical performance depends strongly on carrier composition. Genç and Demirel prepared positively charged pilocarpine hydrochloride nanoparticles using either Eudragit^®^ RS 100 or Gelucire^®^ 44/14 and evaluated the effect of the cationic lipid octadecylamine. Entrapment efficiency varied markedly among formulations, from 31% to 85%, and increasing the octadecylamine concentration reduced drug entrapment; for example, efficiency decreased from 85% in a formulation without octadecylamine to 31% in one containing 0.5% *w*/*v*. Particle size and surface charge were similarly formulation-dependent, and selected autoclaved formulations remained within the nanometer range during 6 months of storage, although changes in particle size, zeta potential, and pH occurred under some storage conditions [50]. Thus, this study primarily demonstrated the sensitivity of nanoparticle properties to formulation composition and processing rather than pharmacological efficacy.

The functional consequence of combining positive surface charge with a mucoadhesive polymeric structure was investigated more directly with chitosan/Carbopol nanoparticles. Pilocarpine loading increased particle diameter from 250 ± 23 to 294 ± 30 nm, while the final formulation retained a positive zeta potential of 55.78 ± 3.41 mV and achieved an encapsulation efficiency of 77 ± 4%. More than 90% of pilocarpine was released from conventional eye drops within 4 h, whereas nanoparticles, liposomes, and gel showed sustained release for up to 24 h, with the nanoparticle formulation providing the slowest release profile. In rabbits, the magnitude and duration of miosis followed the same order—nanoparticles > liposomes > gel > eye drops—and both nanoparticles and liposomes prolonged the miotic response for up to 24 h; the nanoparticle formulation also produced the largest AUC0–24 for miosis [51]. These data concern sustained release and a pharmacodynamic miotic endpoint and should not be interpreted as direct evidence of superior IOP reduction.

Nanomicelles extend this principle through the self-assembly of surfactants or amphiphilic polymers into structures with a hydrophobic core and hydrophilic shell. Their principal rationale in ocular delivery is the solubilization of poorly water-soluble compounds while maintaining a nanoscale formulation capable of interacting with ocular barriers. Reviews of this field emphasize that micellar performance cannot be considered independently of formulation characteristics: particle size and polydispersity, surface charge, encapsulation efficiency, release behavior, and physical stability all influence their suitability for ocular administration. More recent reviews similarly identify polymeric and surfactant nanomicelles as platforms for improving drug solubilization and ocular penetration, while emphasizing that successful clinical translation requires control of formulation characteristics rather than self-assembly alone [52,53,54].

One way to extend micellar residence beyond that obtainable with an eye drop is to incorporate the carrier into a drug-eluting device. Xu et al. embedded mPEG–PLA micelles co-loaded with timolol and latanoprost within contact lenses [55]. In vitro, timolol and latanoprost were released for 144 and 120 h, respectively; in rabbit tear fluid, they remained detectable for 120 and 96 h. Compared with eye drops, tear-fluid pharmacokinetic analysis showed 79.6- and 122.2-fold increases in MRT and study-defined relative bioavailability increases of 2.2- and 7.3-fold for timolol and latanoprost, respectively. In rabbits with elevated IOP, the combination lens maintained IOP reduction for >168 h and achieved a 9.8-fold higher relative pharmacological availability, calculated from the area above the IOP–time curve, than the eye-drop comparator [55]. These results were obtained despite lower drug doses in the lens than in the comparator eye drops but remain evidence from an experimental rabbit model rather than chronic human wear.

Contact-lens incorporation also shows that increased nanoparticle-mediated drug loading does not necessarily translate into slower release. Maulvi et al. used gold nanoparticles either within the contact lens or in the timolol soaking solution and demonstrated increased timolol uptake without significant changes in lens swelling or optical transmittance. However, the gold nanoparticles did not significantly improve the in vitro release-rate profile. Their principal effect emerged in vivo: timolol concentrations in tear fluid were higher than with conventionally soaked lenses, and after 24 h approximately fourfold and twofold increases in drug concentration were observed in the iris–ciliary muscle and conjunctival tissue, respectively. Both the gold-nanoparticle lens and the conventionally soaked lens prolonged IOP reduction for 72 h, with an average decrease of approximately 2 mmHg. Thus, the clearest contribution of the gold nanoparticles in this study was increased drug loading and ocular tissue exposure rather than a major change in sustained-release kinetics [56].

Nanoparticle retention can also be increased by applying an external physical driving force. Kim et al. combined latanoprost-loaded PLGA nanoparticles with noninvasive iontophoresis delivered through skin-attached electrodes and directly compared particles of different sizes. Increasing particle size prolonged drug release but reduced iontophoretic tissue penetration, producing an optimum balance at approximately 300 nm under the tested conditions of 4 mA for 30 min. A single iontophoretic administration of the 300-nm formulation maintained latanoprost exposure in the aqueous humor for up to 12 days, with a drug-concentration AUC of 16,073.9 compared with 675.9 for Xalatan^®^, a >23-fold difference. The IOP-lowering AUC was 4650.9%·h versus 282.7%·h with Xalatan^®^, a >16-fold increase, and IOP reduction persisted for more than 7 days. However, the nanoparticle tablet contained approximately 170–187 μg of latanoprost, whereas a single Xalatan^®^ drop contained 0.175 μg in the study; these AUC ratios therefore reflect both the combined nanoparticle–iontophoresis strategy and markedly different administered drug amounts and should not be interpreted as dose-normalized effect sizes. Corneal microscopy, OCT, and histology did not reveal significant damage under these experimental conditions, although the evaluation remained preclinical and assessed a single treatment strategy [57]. Importantly, these effects cannot be attributed to the PLGA nanoparticles alone because the intervention combined nanoparticle formulation with an external iontophoretic driving force and a distinct administration strategy. The >23-fold increase in drug-exposure AUC and >16-fold increase in IOP-effect AUC therefore represent the performance of the combined nanoparticle–iontophoresis system rather than an isolated estimate of nanoparticle superiority. Particle architecture remained relevant, as particle size influenced the balance between sustained release and tissue penetration, but the relative contributions of the nanocarrier and iontophoresis cannot be disentangled from this study design.

A further extension is to use the same nanoparticle to deliver therapeutics directed at different components of glaucoma pathology. Li et al. developed PEG–PSA nanoparticles co-delivering brinzolamide and miRNA-124 by intravitreal injection. The particles measured 250–400 nm, achieved approximately 90% encapsulation and 6% drug loading, and showed sustained in vitro release from day 1 to day 12. In a microbead-induced ocular-hypertension model, IOP decreased from 22.50 ± 3.89 mmHg on day 3 to 12.83 ± 1.47 mmHg on day 6 after treatment, compared with 22.83 ± 3.31 and 22.17 ± 3.19 mmHg in the PBS and untreated microbead groups, respectively (*p* < 0.01). In a separate optic-nerve-crush model, Tuj-1-positive RGC survival reached 96.09 ± 1.48% versus approximately 84% in both control groups, while RGC apoptosis was 18.50 ± 6.94% versus 43.86 ± 5.31% and 46.12 ± 6.89% (*p* < 0.01) [58]. These experiments provide proof of concept for combining an IOP-lowering agent with a molecular strategy directed toward RGC survival. However, the pressure-lowering and neuroprotective outcomes were demonstrated in separate experimental models, and the findings should therefore not be interpreted as evidence that the formulation modifies glaucomatous neurodegeneration independently of IOP lowering. The approach remains experimental, uses an intravitreal route, and requires further validation in glaucoma models that directly link pressure control, RGC preservation, and functional outcomes.

Finally, nanoparticles can modify drug penetration without encapsulating the hypotensive agent. Nagai et al. prepared magnesium hydroxide nanoparticles with a mean diameter of 71 nm and co-instilled them with dissolved 0.5% timolol. The nanoparticles themselves did not penetrate the cornea, but increased the corneal intercellular-space ratio to 22.1 ± 3.4%, compared with 4.1 ± 1.2% in untreated corneas and 7.9 ± 2.7% with magnesium hydroxide microparticles. Correspondingly, in vitro timolol flux increased from 8.3 ± 2.1 to 12.0 ± 1.1 nmol·cm^−2^·min^−1^, while in vivo aqueous-humor AUC0–90 min increased from 906 ± 168 to 2845 ± 197 μM·min (*p* < 0.05). The combination also produced significantly greater IOP lowering than timolol solution or the corresponding microparticle formulation. Repeated administration of magnesium hydroxide nanoparticles for 14 days produced no observable corneal toxicity, although the authors considered the precise mechanism underlying the permeability enhancement unresolved [59].

Taken together, these systems broaden the role of nanoscale engineering beyond drug encapsulation alone: nanoparticles can serve as mucoadhesive reservoirs, components of sustained-release devices, substrates for physically enhanced delivery, vehicles for simultaneous pharmacological and molecular therapy, or modifiers of corneal drug penetration. This versatility also means that effective ocular delivery depends on the interaction between carrier composition, surface properties, retention, biological barriers, and the final mode of administration rather than on particle size alone. More broadly, rapid ocular clearance, nonspecific biological interactions, and the need to achieve adequate penetration and tissue retention remain important constraints in the design of clinically useful ocular nanocarriers [60].

To facilitate clinically meaningful comparison across heterogeneous delivery strategies, Table 1 summarizes representative nanoengineered systems according to the complete delivery route, final dosage form, experimental model, evidence level, comparator, dosing conditions, follow-up, principal pharmacokinetic or transport and pharmacodynamic findings, safety, and translational limitations. Importantly, the table distinguishes effects attributable predominantly to the nanoscale formulation from those that reflect the performance of the complete delivery system, including the external vehicle, administration route, device, or physical driving force.

The table provides a cross-formulation comparison of representative studies rather than a formal efficacy ranking, because the formulations were evaluated using heterogeneous experimental models, comparators, dosing regimens, and study designs. Where ocular bioavailability or duration was not directly quantified, this is explicitly indicated. Abbreviations: AUC, area under the curve; IOP, intraocular pressure; OCT, optical coherence tomography; PLA, poly(lactic acid); PLGA, poly(lactic-co-glycolic acid).

## 3. Discussion

The evidence reviewed above is interpreted here through a formulation–performance framework rather than as a ranking of nanocarrier classes. The analysis addresses four questions: whether formulation characteristics improve ocular drug exposure; whether increased exposure translates into greater or more durable IOP lowering; whether these relationships remain consistent across drugs, experimental models, and administration routes; and whether the resulting strategies have progressed from preclinical proof of concept to credible human evidence. This distinction is essential because improvements in particle characteristics, drug loading, release, permeability, or ocular exposure do not necessarily predict therapeutic performance.

### 3.1. Formulation Architecture and Ocular Drug Exposure

The central finding of this review is not that one nanoparticle class is uniformly superior to conventional glaucoma therapy, but that nanoscale engineering can modify several determinants of ocular drug disposition that are difficult to control with conventional eye drops. Topically administered drugs are rapidly removed from the ocular surface, and typically less than 5% of the applied dose reaches intraocular tissues because of precorneal elimination and subsequent epithelial barriers [12,13,14]. Nanocarriers can modify drug solubility, stability, surface interaction, penetration, and release kinetics [15,19,20]. This pharmacological rationale is supported by the available preclinical evidence: a meta-analysis of 16 animal studies of topical particulate antiglaucoma systems reported a pooled improvement in pharmacodynamic effect of 7.987 (95% CI, 5.334–10.639; *p* < 0.001) compared with drug solutions [17]. However, heterogeneity was very high (*I*^2^ = 91%), and no significant advantage of lipid over non-lipid carriers emerged, indicating that performance cannot be predicted simply from nanocarrier class.

This heterogeneity is consistent with the individual studies reviewed here, which repeatedly show that ocular performance is determined by the interaction among carrier architecture, drug properties, surface characteristics, and administration route rather than by particle size alone. Indeed, optimization of one formulation parameter does not necessarily optimize the final pharmacological response. In the surface-modified SLN study, for example, timolol release and permeation became more sustained, but the free drug still exhibited a higher permeability coefficient than the nanoparticle formulations [25]. With hollow PLA nanoparticles, increasing shell thickness prolonged pilocarpine release while progressively reducing encapsulation efficiency, and an intermediate shell thickness of approximately 40 nm provided the most useful balance between drug incorporation and release duration [26]. Similarly, acetazolamide niosomes with the highest entrapment efficiency were not those producing the best pharmacodynamic performance, because cholesterol content and lamellarity simultaneously altered drug retention and release [39]. These observations argue against using encapsulation efficiency, particle size, or any other isolated physicochemical parameter as a surrogate for therapeutic performance. The lipid-based studies provide a particularly clear example of this distinction. In the timolol SLN study, sustained permeation was achieved despite lower instantaneous permeability than with free drug [25]; in the brimonidine NLC/SLN comparison, prolonged IOP lowering occurred despite lower total corneal permeation than with free brimonidine preparations [32]; conversely, TPGS-modified brinzolamide liposomes produced both markedly greater apparent transcorneal permeability and a longer pharmacodynamic effect than the commercial comparator [34]. These findings show that permeability coefficient, cumulative tissue delivery, ocular residence, and duration of IOP lowering cannot be treated as interchangeable markers of formulation performance. Rather, the therapeutic effect reflects how the formulation reshapes the complete ocular concentration–time profile.

### 3.2. From Ocular Exposure to IOP-Lowering Efficacy

There is a similar relation between ocular exposure and pharmacodynamic efficacy. It is with an appropriate concentration-time profile at the target tissue that increased drug delivery becomes relevant. It is also clear that greater exposure does not on its own prove superior IOP lowering. This distinction was particularly clear in the lipid–DNA travoprost study. Ocular drug delivery was approximately fourfold greater 4 h after administration. However, 50% of the nanoparticle-treated eyes versus 33% of the free-travoprost-treated eyes responded. Furthermore, the mean relative IOP reductions amongst responding eyes were 30% versus 17%. In addition, the between-group difference remained statistically inconclusive (*p* = 0.1) [38]. Consequently, the study shows enhanced delivery to the eye, but not established pharmacodynamic superiority. In contrast, the iontophoretic PLGA system achieved aqueous-humor drug exposure more than 23-fold greater than Xalatan^®^ and an IOP-effect AUC more than 16-fold greater, resulting in over 7 days of IOP reduction [57]. The effect resulted from a combined nanoparticle–iontophoresis intervention; nanoparticle size affected release and penetration into tissue, while the external electrical driving force and administration strategy also contributed. Thus, the nanoparticle architecture alone is not responsible for the strength of the effect. These studies indicate that factors like nanoparticle architecture, formulation vehicle, administration route, and external delivery forces may independently or interactively contribute to the final pharmacological response. Moreover, they show that we cannot interpret increased penetration or prolonged exposure alone as evidence of nanoparticle superiority.

The distinction between formulation performance and pharmacodynamic benefit is also relevant when nanoparticles are incorporated into larger delivery devices. Micelle-loaded contact lenses increased the mean residence time of timolol and latanoprost by 79.6- and 122.2-fold and their relative bioavailability by 2.2- and 7.3-fold, respectively, while maintaining IOP reduction for more than 168 h in rabbits [55]. However, incorporation of gold nanoparticles into timolol-loaded contact lenses illustrates that different formulation improvements may become dissociated: gold nanoparticles increased drug uptake and ocular tissue deposition, including approximately fourfold greater timolol concentration in the iris–ciliary muscle after 24 h, but did not significantly improve the in vitro release-rate profile [56]. Accordingly, nanoparticle-mediated increases in loading, residence, or tissue accumulation should not automatically be interpreted as evidence of superior sustained efficacy.

#### IOP-Lowering Nanodelivery, Glaucomatous Neurodegeneration, and Disease-Modifying Nanomedicine

It should be noted that there is an important differentiation between nanoengineered delivery that can improve control of IOP and nanomedicine designed to alter the neurodegenerative component of glaucoma directly. Almost all studies included in this review belong to the first category. That is to say, nanoparticles alter the solubility, residence, penetration, ocular exposure, or release of already-established IOP-lowering agents to enhance or prolong their pharmacological action. The relevance of these strategies to glaucoma remains high because IOP, or intraocular pressure, is the main modifiable risk factor. Moreover, control of IOP is a primary focus of current therapy. However, better ocular pharmacokinetics or sustained IOP lowering per se should not be construed or claimed to confer direct neuroprotection or disease modification.

Nanoparticle systems may provide advantages beyond simple IOP reduction, but these advantages should be classified according to the biological objective being pursued. The hollow ceria nanoparticle [27] was multifunctional. Apart from pilocarpine delivery, ceria showed experimentally proven antioxidant and anti-inflammatory activity, so the intended aim was not only drug-release control but also biological activity. By contrast, the brinzolamide/miRNA-124 PEG–PSA system [58] represents a combined IOP-lowering and RGC-directed strategy; a conventional hypotensive agent is coupled with molecular cargo intended to improve RGC survival. The above-mentioned strategies are fundamentally different from most of the formulations reviewed here, whose aim is the sustained delivery of established IOP-lowering drugs. These findings are also still preclinical proof of concept and should not be taken to mean that antioxidant, anti-inflammatory, or RGC-preserving nanomedicine has established clinical efficacy in glaucoma.

Consequently, the nanoengineered systems considered in this review can, from a therapeutic-intent perspective, be divided into several classes. Nanotechnology is mainly used in IOP-lowering delivery systems to enhance the solubility, retention, penetration, release, or duration of established ocular hypotensive agents. Another category includes multifunctional systems, wherein the carrier or the co-delivered cargo is intended to provide biological effects other than pressure lowering, such as antioxidant or anti-inflammatory activity. The third, much less mature group comprises neuroprotective or RGC-directed strategies that aim to preserve retinal ganglion cells or their axons by mechanisms not reducible to IOP lowering. Neuroregenerative strategies should be distinguished from neuroprotection, as regeneration requires restoration or regrowth of damaged neural structures rather than preservation of surviving cells. Neuroregenerative efficacy has not been convincingly demonstrated in the primary nanoengineered glaucoma studies synthesized here.

The same distinction applies to the biological processes that underlie glaucomatous injury. A rise in intraocular pressure (IOP) can trigger or exacerbate mechanical and metabolic stress, but the downstream neurodegenerative phenotype represents progressive dysfunction and loss of retinal ganglion cells (RGCs) and their axons. Consequently, optic-nerve damage refers to the structural and axonal manifestation of the neurodegenerative process rather than a direct surrogate for IOP itself.

Neuroinflammation, oxidative stress, mitochondrial dysfunction, and excitotoxicity are interacting molecular and cellular mechanisms that may contribute to RGC vulnerability, axonal dysfunction, and cell death. Thus, while a formulation that reduces IOP acts primarily on an established upstream modifiable risk factor, a neuroprotective formulation must act on one or more of these downstream injury pathways and show preservation of neural structure or function.

Disease-modifying or neuroprotective nanomedicine has a different therapeutic objective. Such strategies would need to preserve retinal ganglion cells, axons, optic-nerve structure, or visual function by mechanisms not solely reducible to IOP lowering, rather than primarily improving delivery of a conventional hypotensive agent. The brinzolamide/miRNA-124 nanoparticle system [58] illustrates this conceptual difference. Brinzolamide produced an IOP-lowering effect in a microbead-induced ocular-hypertension model; however, RGC survival and apoptosis were assessed separately in an optic-nerve-crush model. As a result, the study offers experimental support for a strategy involving pharmacological and molecular delivery, but it does not establish disease modification in glaucoma or neuroprotection independent of pressure lowering. The translational requirements of these two therapeutic pathways differ. An initial evaluation of ocular pharmacokinetics, sustained IOP control, tolerability, and dosing burden is appropriate for IOP-lowering nanodelivery, and its final evaluation can include comparative clinical efficacy against established hypotensive therapy. A neuroprotective strategy requires convincing target engagement together with structural and functional evidence of RGC or optic-nerve preservation, appropriate glaucoma models, durability of benefit, and eventually demonstration that neuroprotection exceeds what can be explained by IOP lowering alone. Long-term visual-field preservation or another validated functional outcome will ultimately be required before such an approach could be considered disease modifying in clinical glaucoma. Thus, prolonged IOP lowering and neuroprotection should be regarded as complementary but not equivalent therapeutic endpoints.

Neuroinflammation, oxidative stress, mitochondrial dysfunction, and excitotoxicity are biologically relevant candidate targets. However, the detailed primary evidence synthesized here does not directly address all these mechanisms. Among the nanoengineered systems included in the review, direct experimental evidence beyond IOP lowering remains limited and heterogeneous.

### 3.3. Reproducibility Across Formulations, Drugs, and Experimental Models

The diversity of these results also raises the question of whether formulation–performance relationships are reproducible across drugs, experimental models, and administration routes, and makes broad comparisons between nominal carrier classes of limited value. Lipid nanoparticles, polymeric nanoparticles, niosomes, nanomicelles, nanocrystals, inorganic nanoparticles, and hybrid systems each encompass formulations with markedly different dimensions, surface charge, drug-loading mechanisms, release profiles, and biological interactions. Even within the same category, formulation changes can substantially alter performance. Brimonidine-loaded NLCs and SLNs, for example, differed in the onset and magnitude of IOP reduction, although both remained active for more than 24 h [32], whereas modification of conventional niosomal delivery through a proniosomal precursor produced a 7.90-fold increase in mean residence time and maintained measurable IOP lowering at 24 h [33]. For acetazolamide niosomes, surfactant type, cholesterol ratio, and preparation method all changed entrapment and release [39]. The clinically relevant unit of comparison is therefore the complete formulation rather than the generic nanocarrier label. This distinction is particularly important across the operational categories defined in this review: carrier-based nanoparticles, drug nanocrystals, permeability modifiers, multiscale formulations, and nanoparticle-integrated devices should not be interpreted as directly interchangeable ‘nanoparticle platforms,’ because the observed pharmacological effect may arise from different combinations of nanoscale structure, formulation vehicle, administration route, and device contribution.

### 3.4. Human Evidence, Patient-Centered Outcomes, and Clinical Applicability

Against this extensive preclinical literature, human evidence remains limited. Wong et al. evaluated a single subconjunctival administration of liposomal latanoprost in an open-label, non-comparative study of only six subjects with ocular hypertension or primary open-angle glaucoma [28]. IOP lowering persisted for up to 3 months, providing an important first-in-human demonstration of prolonged pharmacological activity, but the design and sample size preclude conclusions regarding comparative efficacy or long-term safety. Another human study included in this review evaluated 3% dorzolamide/γ-cyclodextrin nanoparticle eye drops in 17 subjects using a randomized crossover design. Under the specific conditions of this small crossover study, a single nanoparticle dose produced a broadly similar short-term IOP profile to three administrations of commercial dorzolamide, while burning sensation was significantly lower with the nanoparticle formulation [48]. Importantly, the absence of a statistically significant difference between treatments does not establish equivalence or non-inferiority, and the study was not interpreted as demonstrating that one nanoparticle dose is therapeutically equivalent to three conventional doses. These studies establish human feasibility, but they remain too small and short to determine whether nanoparticle-mediated delivery improves sustained disease control, adherence, ocular-surface outcomes, or functional glaucoma progression.

**Figure 2:**
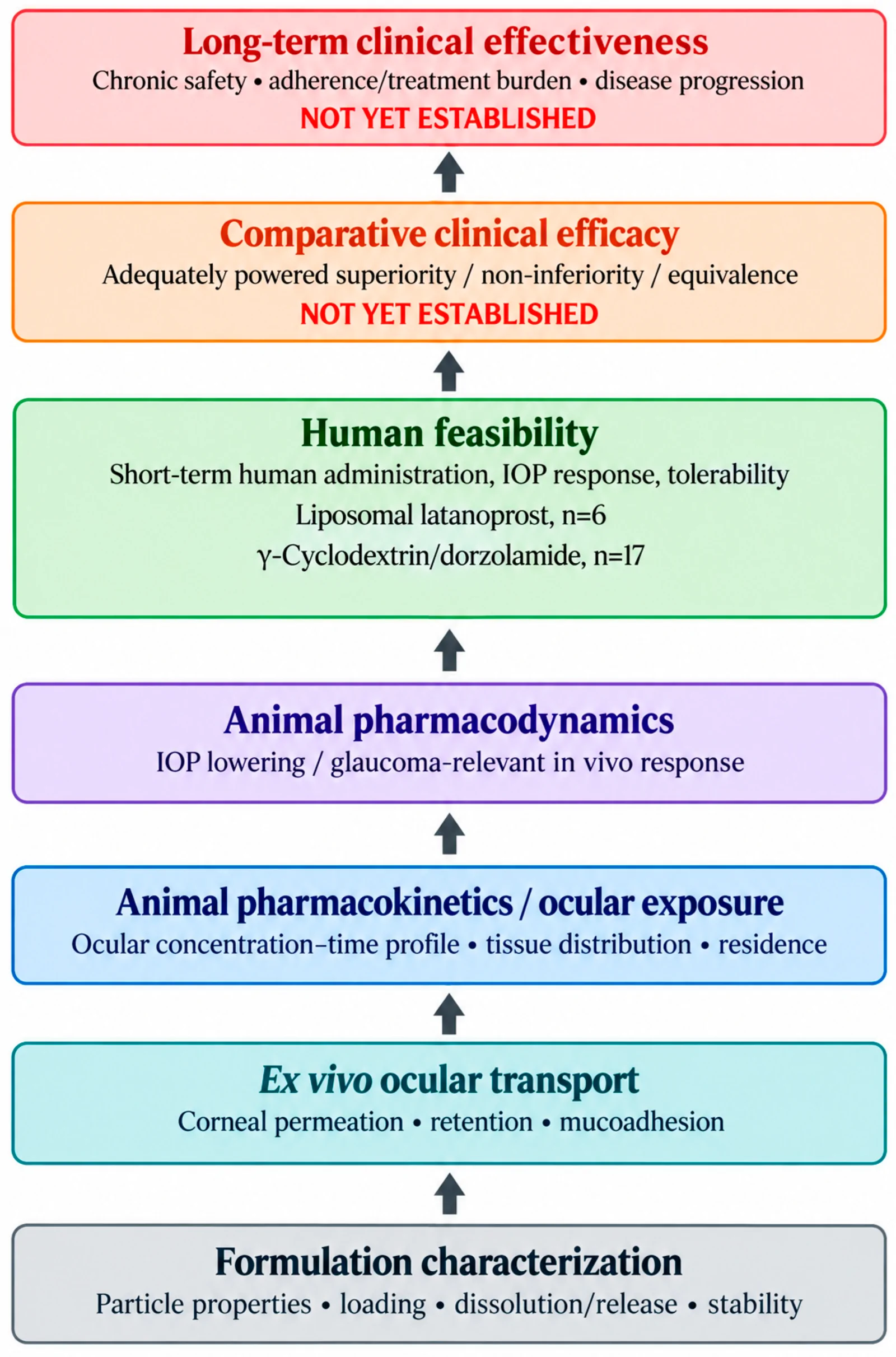
Evidence hierarchy for interpretation of nanoengineered glaucoma drug-delivery studies. Evidence progresses from formulation characterization and ex vivo ocular transport through animal pharmacokinetics and pharmacodynamics to human feasibility, comparative clinical efficacy, and long-term clinical effectiveness. The nanoparticle-specific human evidence identified in this review currently reaches the human-feasibility stage through the small studies of subconjunctival liposomal latanoprost [28] and topical γ-cyclodextrin/dorzolamide [48]. Adequately powered comparative clinical efficacy, long-term safety, adherence benefit, and effects on glaucoma progression remain unestablished. No nanoparticle-specific glaucoma formulation identified in this review has reached approved routine clinical availability.

Because the reviewed literature spans markedly different experimental levels, the evidentiary meaning and principal limitations of each level are defined in Table 2 and represented graphically in Figure 2.

Given the limited amount of clinical evidence, all human studies identified in this review are summarized separately in Table 3.

To facilitate study-level comparison across the heterogeneous evidence base, the principal design, formulation, pharmacokinetic, pharmacodynamic, tolerability, and methodological characteristics of the primary studies are summarized in Table 4.

A crucial limitation of this synthesis is that no formal risk-of-bias assessment was undertaken. Since the included evidence stems from fundamentally different experimental designs, their apparent consistency across studies should not be taken to mean equivalent methodological robustness. Many of the biggest reported improvements in ocular exposure or duration of action come from exploratory preclinical studies with small or incompletely reported sample sizes, short follow-up, heterogeneous or non-standardized comparators, and, in some cases, interventions in which the nanocarrier and delivery device or method of administration were modified at the same time. Many of these attributes compromise internal validity, as well as the ability to attribute effects specifically to the nanoengineered component. Consequently, here, substantial relative enhancements in pharmacokinetic exposure or pharmacodynamic duration are regarded as design-specific proof of concept rather than as directly comparable estimates of treatment superiority. When the findings are supported by an appropriate comparator, clearly characterized dosing and follow-up, and consistent pharmacokinetic and pharmacodynamic evidence, confidence is therefore greatest. By contrast, conclusions based on formulation-only, ex vivo, small-animal, or preliminary human studies remain necessarily more provisional. This limited clinical evidence should be borne in mind when interpreting the sometimes striking durations reported in the experimental studies. The meta-analysis itself was limited to animal studies and showed substantial between-study heterogeneity in experimental conditions, measurements, and sample sizes [17]. In the context of the animal species and disease model, administration route, dose and dosing regimen, comparator, and follow-up period used in each study, reported durations of IOP lowering should not be considered directly comparable estimates of treatment durability across formulations. More specifically, duration must be interpreted in the context of the pharmacokinetic constraints imposed by each route: for example, a topical formulation has to overcome precorneal clearance and epithelial barriers, while periocular or intraocular administration bypasses some barriers by establishing a depot closer to the target tissues, and device-assisted systems can prolong exposure via an external reservoir or physical driving force. A longer effect obtained through a more invasive or device-dependent route does not necessarily indicate superior delivery performance.

Within the studies reviewed here, experimental efficacy ranged from hours with some topical formulations to several days or weeks with iontophoretic, intracameral, or subconjunctival systems. Such results demonstrate what the delivery technologies can achieve experimentally, but they should not be converted directly into expectations regarding human dosing intervals. Evidence of prolonged exposure in an animal model is therefore better regarded as a basis for clinical investigation than as evidence of an established therapeutic advantage.

An important translational goal is to reduce the frequency of treatment, but treatment burden and adherence cannot be presumed directly from the duration of pharmacodynamic effect. There are different multifactorial reasons why glaucoma medication adherence is poor. Actually, patients report forgetfulness, difficulty following medication schedules, difficulty instilling eye drops, limited self-management ability, and combinations of these [7,8,9]. A formulation that prolongs pharmacological activity from hours to days or weeks may reduce dosing frequency. However, whether this results in better persistence or adherence requires specific clinical testing. The appropriate endpoint is consequently not merely the interval between doses, but the total burden imposed by the entire treatment regimen.

Different delivery platforms have different burdens. A long-acting topical formulation may permit a reduction in daily administrations while still allowing familiar self-administration. However, a subconjunctival depot requires an office-based procedure and acceptance of repeated injections if retreatment is necessary [28,46]. Contact-lens-based delivery does not require repeated drop instillation but does introduce the requirements of lens insertion, removal or replacement, handling, comfort, and maintenance [55,56]. Iontophoretic systems lessen the need for conventional instillation; however, they do rely on a device and treatment procedure [57]. As such, a longer pharmacodynamic duration should not simply be associated with a lower burden to the patient; potential benefit should be weighed against procedure frequency, comfort, handling needs, caregiver dependence, healthcare-resource use, accessibility, and cost.

The probable impact of patient characteristics on the practical value of each approach should be assessed prospectively and not assumed from preclinical performance. In particular, evaluation of contact-lens-based delivery is required in the population for whom chronic glaucoma therapy is intended. Elderly patients suffering from ocular-surface disease, reduced manual dexterity, cognitive impairment, difficulty manipulating a lens, or dependence on caregivers may have a different balance of benefit and burden than that observed in experimental animal models or in younger contact-lens users. Conversely, some patients who encounter challenges in using eye drops may derive considerable benefit if a sustained system can be used comfortably and reliably. As such, future human studies should also assess patient acceptance, treatment preference, comfort, handling feasibility, caregiver involvement, accessibility, cost and healthcare-resource utilization, treatment persistence, and vision-related or treatment-related quality of life in addition to pharmacological efficacy.

Ocular-surface tolerability represents a second clinically relevant endpoint. Chronic topical glaucoma treatment, particularly with preserved formulations, is associated with ocular-surface abnormalities [10,11]. The recent hyaluronic-acid-coated latanoprost liposomes are notable because their formulation strategy addressed drug delivery and ocular-surface compatibility simultaneously rather than treating tolerability as a secondary endpoint [30,31]. A single dose of LAT-HA-LIP prolonged the hypotensive response to 48 h compared with 24 h for the marketed comparator and produced 2.97-fold greater relative ocular bioavailability [30]. With repeated administration, tear osmolarity, tear breakup time, Schirmer testing, and fluorescein staining remained broadly within physiological ranges during the reported monitoring period, while daily treatment produced mean IOP reductions of 2.51–3.88 mmHg between days 5 and 15 [31]. However, efficacy was evaluated for only 15 days, and the authors explicitly identified substantially longer studies as necessary to establish chronic safety and efficacy. This is particularly relevant for glaucoma, where tolerability must be demonstrated under repeated exposure rather than inferred from short-term absence of irritation.

When ordered by evidence maturity, rather than the size of individual experimental effects, no one delivery technology appears as the overall ‘best’ strategy. The highest existing evidence level at the moment is human feasibility. The clearest evidence of prolonged IOP lowering, mediated by nanoparticles, in humans comes from an open-label, non-comparative pilot study involving only six subjects [28]. This evidence involves subconjunctival liposomal latanoprost. The extra benefit of using topical γ-cyclodextrin/dorzolamide is that it has a non-invasive mode of administration and has been evaluated in a randomized crossover human study. However, the 17-subject, 24-h study only establishes preliminary short-term feasibility and tolerability and does not establish equivalence, durable clinical effectiveness, or reduced long-term treatment burden [48]. Consequently, neither of the systems currently provides sufficient evidence for comparative effectiveness, long-term safety, adherence benefit, or prevention of glaucoma progression.

Below this human-feasibility level, various platforms have presented strong preclinical pharmacodynamic evidence that is not directly comparable. Topical lipid-based and polymeric formulations have demonstrated prolonged ocular exposure and IOP lowering without an invasive procedure [30,31,37,43,44]. However, their efficacy is dependent on formulations and vehicles and has not been tested in adequately powered human studies. Although contact-lens systems can hold a non-injectable ocular drug reservoir for days on end, their seemingly attractive benefit arises from the complete nanocarrier–device system, whose chronic human wear has not been established [55,56]. The sustained effects of iontophoretic PLGA nanoparticles [57] and intracameral hollow PLA nanoparticles [26] stem from the different delivery systems employed: an externally assisted iontophoretic route and an invasive intraocular route, respectively, so they should not be compared directly with those from topical or contact-lens systems. Other systems provide mainly mechanistic or pharmacokinetic evidence. Surface-modified timolol SLNs prolonged permeation, but in vivo IOP efficacy was not demonstrated [25]. Chitosan-coated dorzolamide nanoparticles increased ex vivo mucoadhesion and corneal transport, but no in vivo IOP experiment was performed [45]. The difference between these levels is evidenced by lipid–DNA travoprost nanoparticles, because markedly increased ocular delivery did not establish statistically significant pharmacodynamic superiority [38]. As such, fitness for purpose for the management of chronic glaucoma depends not on the longest experimental duration or biggest exposure ratio alone, but on the combination of demonstrated IOP control, route acceptability, repeatability, ocular safety, treatment burden, manufacturability, and maturity of clinical evidence. This evidence-maturity framework is summarized in Table 1.

#### 3.4.1. Clinically Relevant Comparators and Outcome Selection

The choice of comparator also critically influences the interpretation of preclinical efficacy. Demonstrating that a nanoengineered formulation generates greater exposure or extended IOP lowering than an aqueous drug solution establishes experimental proof of concept, but does not necessarily demonstrate an advantage over the marketed formulation commonly used in practice. Translational studies should preferentially use clinically relevant active comparators administered according to their established dosing regimen. In the event that differences exist between the experimental and control groups in drug dose, concentration, frequency of administration, or route of administration, such differences should be matched where appropriate or justified explicitly, and the treatment effect should not be attributed only to the nanoengineered component.

The comparator regimen should reflect the intended clinical role of the new delivery system rather than just a match of individual administrations. For instance, a long-acting injection or device-based treatment should not be assessed only against a single administration of an eye drop if its intended advantage is the replacement of repeated treatment over days, weeks, or months. The relevant clinical comparison is the total therapeutic strategy over the proposed therapeutic interval, including dose, frequency of retreatment, administration burden, safety, and resources needed. This difference matters especially for invasive or device-dependent platforms, where prolonged activity may have procedural or handling burdens that conventional topical treatment does not carry.

The focus of outcome selection must go beyond just the maximal or mean IOP reduction. In order to support translation, standardized IOP-measurement procedures, prespecified pharmacodynamic endpoints, appropriate baseline and time-course assessment, and follow-up durations matching the intended dosing interval should be used in preclinical studies. Moreover, human studies should also evaluate diurnal IOP control, persistence of the treatment effect, requirement for rescue or retreatment, ocular-surface effects, local and systemic adverse effects, treatment discontinuation, adherence or persistence, patient-reported treatment burden, comfort, quality of life, and healthcare-resource requirements. The aim of chronic glaucoma treatment is sustained and acceptable disease control over time rather than maximizing a single pharmacokinetic parameter or short-term IOP measurement.

#### 3.4.2. Route-Specific Interpretation of Treatment Duration

The length of time IOP is lowered should be interpreted through route-specific delivery considerations rather than as a generic cross-platform index of durability. Topical formulations encounter various obstacles, such as precorneal clearance and epithelial barriers. Periocular administration can bypass much of the precorneal compartment and set up a local depot. Intracameral and intravitreal administration place the therapeutic system directly in intraocular compartments. Contact-lens-based platforms establish an external ocular-surface reservoir, and iontophoresis adds an external physical driving force to facilitate tissue delivery. As such, these methods solve fundamentally different pharmacokinetic issues, and the persistence of a reduction in IOP for a few hours or days cannot be compared independently of the means by which that duration was achieved. The choice of route determines which anatomical barriers are relevant. Topical systems must overcome tear-film clearance and epithelial penetration, and periocular systems bypass precorneal loss while still depending on transscleral transport and downstream tissue clearance. Intracameral delivery bypasses ocular-surface entry barriers, while intravitreal delivery shifts the principal delivery constraints to vitreous distribution and retinal access.

To achieve prolonged activity with topical formulations, drug delivery must overcome barriers such as tear turnover, drainage, and corneal penetration. According to previous studies, the topical administration of LAT-HA-LIP exhibits a 48-h effect [30]. Additionally, selected liquid-crystalline travoprost formulations show an activity duration of up to 72 h [37]. However, it is important to interpret these results with recognition of the constraints associated with a non-invasive ocular-surface route. The translational value of these formulations depends not only on pharmacodynamic duration but also on repeated-use tolerability, ease of administration, dosing frequency, and performance relative to established topical therapy.

Periocular and intraocular administration can change these limitations significantly. Subconjunctival administration of liposomal latanoprost [28] and brinzolamide-loaded PLGA nanoparticles [46] results in a local depot that may bypass rapid precorneal clearance. However, the prolonged effects must be balanced against the injection burden as well as local tissue response, depot persistence and clearance, retreatment frequency, and feasibility of repeated administration. Also, after intracameral administration, hollow PLA nanoparticles showed IOP control for over 56 days in an experimental model [26]. However, this duration was achieved by directly placing the nanoparticles within the anterior chamber. Therefore, we cannot interpret this effect as directly superior to shorter effects obtained through topical administration. Its clinical relevance also depends on how invasive the procedure is, how long the treatment persists in the eye, whether the corneal endothelium is harmed, and whether retreatment is acceptable and safe.

Intravitreal delivery is a separate but important translational pathway. The therapeutic goal of the brinzolamide/miRNA-124 nanoparticle system and its tissue exposure are fundamentally different from those of topical or periocular IOP-lowering formulations. The brinzolamide/miRNA-124 nanoparticle system [58] was administered intravitreally to act on both pharmacological and molecular targets. Because prolonged exposure relies on an external drug reservoir in contact with the ocular surface, contact-lens-based systems are also different. The durations reported with micelle- or nanoparticle-loaded lenses [55,56] must be interpreted together with lens wear, replacement, handling, comfort, and chronic ocular-surface compatibility.

The approach is one of whole-system intervention, not just the effect of nanoparticles. The >7-day IOP-lowering effect achieved with PLGA nanoparticles delivered by iontophoresis [57] was dependent on nanoparticle properties and an electrical driving force applied externally. The clinically meaningful measure of durability is not simply the duration of IOP lowering, but the duration achieved relative to the intervention required, dose and comparator conditions, invasiveness, repeatability, retreatment requirements, route-specific adverse effects, and patient and healthcare burden. Most importantly, suitability for chronic treatment is a fundamental criterion. Thus, a >7-day effect after iontophoresis or a >56-day effect after intracameral injection should not be directly compared with a 48- or 72-h effect after topical administration. Each such finding needs to be interpreted in light of the entire route-specific treatment strategy.

### 3.5. Translational Bottlenecks, Long-Term Safety, and Manufacturing Requirements

#### 3.5.1. Why Preclinical Success Rarely Translates into a Clinically Mature Product

A significant gap exists between the extensive preclinical investigation of nanoparticle-based glaucoma therapeutics and the extremely limited testing of these systems in humans. It is unlikely that this gap reflects a single translational barrier. Advancement seems contingent on the successful alignment of many interdependent requirements: a reproducible formulation; a plausible mechanistic relationship between drug release and ocular exposure; translation of increased exposure into meaningful pharmacodynamic benefit; an administration route amenable to repeated chronic treatment; acceptable long-term ocular and systemic safety; scalable and sterilizable manufacture; adequate storage stability; and a clinically meaningful advantage over established glaucoma therapy [19,20,23,60]. Failure at any one of these stages may prevent an experimentally promising system from becoming a clinically useful product.

The studies that we review here provide direct evidence of this. Subconjunctival PLGA brinzolamide nanoparticle formulations were effective in lowering IOP for approximately 10 days despite much longer in vitro release. This shows that longer release in vitro is not a predictor of longer in vivo pharmacodynamic persistence [46]. On the other hand, lipid–DNA travoprost nanoparticles showed substantially greater ocular drug delivery, exhibiting about fourfold greater delivery at 4 h, although they did not establish statistically significant superiority in IOP lowering [38]. These examples show that two forms of surrogate reasoning may lead to translational failure, namely that prolonged in vitro release does not predict prolonged therapeutic effect and that increased tissue exposure does not predict superior pharmacodynamic efficacy.

A framework that can be considered useful is thus progression through five connected requirements. First, one must generate a reproducible and mechanistically interpretable change in drug disposition, as opposed to just favorable physicochemical metrics. Second, this change must demonstrate reproducible pharmacokinetic and pharmacodynamic benefits in biologically relevant models. Moreover, the entire dosage form and route of administration should continue to be acceptable for repeated use in a chronic disease. The formulation should retain its critical characteristics during scale-up, sterilization, storage, and batch-to-batch manufacture. The final product should show a clinically meaningful benefit over established therapy, such as better sustained IOP control, tolerability, treatment burden, or another patient-relevant outcome. A large increase in permeability, exposure, and/or experimental duration is insufficient by itself to demonstrate translational viability.

The final requirement is an important one in glaucoma because new delivery systems are developed in the presence of already existing and effective pharmacological treatments. Any increase in formulation complexity, procedural burden, manufacturing cost, or invasiveness must be justified by a clinically relevant benefit and not by superior laboratory performance alone. The entire balance of efficacy, safety, route acceptability, reproducibility, manufacturability, stability, and treatment burden, rather than a single nanoparticle characteristic, determines commercial and clinical viability.

#### 3.5.2. Long-Term Safety and Route-Specific Risk

Long-term safety requires a substantially different evidentiary standard from short-term ocular tolerability. The absence of acute irritation, corneal staining, or histological damage after a single administration cannot establish safety for a chronic disease in which treatment may continue for years or decades. This distinction is particularly important when a formulation is designed specifically to prolong ocular residence, because increased persistence may also prolong exposure of ocular tissues to the carrier, surfactants, degradation products, or other formulation components. The acetazolamide niosome study illustrates this point: after 40 days of daily administration, slight stromal edema was observed and interpreted as reversible [39]. Such a finding should not be reduced to a binary designation of the system as ‘safe,’ but instead interpreted according to the tissue involved, duration of exposure, reversibility, and intended clinical dosing schedule.

Chronic safety assessments should not only include typical irritation testing, but also consider the entire ocular disposition and fate of the delivery system. Repeated exposure could affect various parameters such as ocular-surface integrity, intraocular biodistribution, persistence or accumulation of the carrier and its components, clearance pathways, identity and biological activity of degradation products, local and systemic exposure, inflammatory or immune responses, and tissue-specific effects on the cornea, corneal endothelium, conjunctiva, sclera, iris and ciliary body, retina, and optic nerve. Studies that use repeated administration are especially important because biological responses to cumulative exposure may not be similar to those after a single dose. Because their degradation, persistence, and clearance mechanisms may differ considerably, these issues are particularly relevant to biodegradable polymers, surfactant-containing vesicular systems, inorganic nanoparticles, and hybrid carriers. Safety evaluation must also be explicitly linked to the cell populations and tissues that are exposed by the intended delivery strategy. As repeated exposure necessitates an evaluation of corneal and conjunctival epithelial viability, topical formulations must meet these requirements. Moreover, systems targeted to the conventional outflow pathway will need a similar assessment of function in trabecular-meshwork cells. Lastly, formulations for the posterior segment or those intended to be neuroprotective require evaluation of retinal cells, RGCs, and optic-nerve tissues. Besides direct cytotoxicity, chronic studies should evaluate inflammatory responses, formulation-induced oxidative stress, persistence or accumulation of poorly cleared carrier material, and biological activity of degradation products. Short-term lack of irritation or histological damage cannot replace chronic safety evaluation specific to route and tissue type [19,20,23,60].

The safety assessment must also match the intended route of administration. When it comes to topical formulations, repeated exposure of the corneal and conjunctival epithelium and the long-term effects of the complete vehicle are critical. Subconjunctival depot evaluation should assess local tissue responses, depot persistence and clearance, conjunctival and scleral changes, and the effects of repeated injections. Intracameral systems must also be specifically considered for anterior-segment distribution and corneal endothelial safety, while intravitreal platforms require evaluation of intraocular persistence, retinal exposure, inflammatory responses, and posterior-segment clearance. Because prolonged drug and nanocarrier exposure occur together with extended lens–ocular-surface interaction, contact-lens-based systems introduce a different safety context. Therefore, the safety profile generated for one route cannot be transferred to another formulation or administration strategy on an automatic basis.

The existing preclinical studies already show why this route- and tissue-specific interpretation is necessary. The formulations in the subconjunctival PLGA brinzolamide study were not entirely free of tissue findings. These findings included transient subconjunctival bleb and vascularization. One formulation also resulted in iris vascular congestion. Furthermore, mild reversible retinal degeneration was seen with another formulation [46]. These findings do not establish unacceptable toxicity. However, it is clear that sustained pharmacodynamic action must be interpreted together with biodistribution, tissue response, reversibility, and repeated-dose safety, not just the duration of IOP lowering. As a result, clinical translation necessitates evidence of acceptable tolerance of the complete delivery system over an exposure period and dosing schedule relevant to lifelong glaucoma treatment.

#### 3.5.3. Manufacturing Scale-Up, Sterilization, Storage Stability, Reproducibility, and Regulatory Requirements

Another factor determining translation is whether the experimental formulation can be produced as a reproducible pharmaceutical product through a manufacturing process without altering the attributes that determine ocular exposure and pharmacodynamic performance. The scale-up process must control the nominal composition as well as particle-size distribution and polydispersity, surface charge and other relevant surface characteristics, drug loading and encapsulation state, propensity to aggregate, and release kinetics [20,23,29,53,54,60]. These properties rely on each other. Altering the mixing, homogenization, solvent removal, drying, or other processing conditions can lead to changes in particle architecture; drug distribution within the carrier; the fraction of free or non-encapsulated drug; and, subsequently, release and ocular disposition. The entire formulation should demonstrate batch-to-batch reproducibility, not just maintenance of the drug concentration.

The characteristics of chemical and microbiological quality add extra requirements. When production is scaled up from the laboratory level to larger batches, residual solvents, degradation products, formulation-related impurities, free or non-encapsulated drug, and changes in excipient composition may become especially significant. Ophthalmic formulations should stay sterile, with adequate control of microbial contamination and endotoxin burden, while aggregation and precipitation must stay under control during manufacture and storage. As a result, stability testing should not only cover the preservation of drug content, but also the determination of particle-size distribution, polydispersity, surface characteristics, encapsulation state, aggregation, and drug release behavior over the proposed shelf life under the proposed storage conditions [20,23,29,53,54,60]. A formulation that keeps its nominal drug concentration but undergoes particle growth, drug leakage, aggregation, or a change in release profile cannot be said to be pharmaceutically or pharmacologically equivalent to the experimental preparation.

The sterilization process may alter the structure of the nanoscale product. During autoclaving, the formulation is subjected to heat and pressure, which can destabilize a polymeric matrix, lipid organization, or carrier–drug interactions. Irradiation could alter the structure of a polymer or nanomaterial and thereby modify drug retention or release. Membrane filtration may not be applicable if the dimensions of particles approach or exceed the effective pore size [20]. The sterilization process should not be chosen independently of the formulation architecture. Aseptic manufacture may be necessary when terminal sterilization changes important product characteristics or is technically not feasible. This imposes additional requirements for process control and reproducibility [29,53,54]. Successful sterilization should not only be microbiologically acceptable but should also retain those physicochemical and release properties responsible for the intended ocular performance.

The importance of these translational challenges varies from one nanocarrier class to another. Lipid-based nanoparticles require special consideration in terms of lipid organization during storage and processing. When there is a change in crystallinity or physical state, there can be drug expulsion or leakage, nanostructure aggregation, and changes in release behavior. It is important to validate thermal processing and sterilization. Polymeric nanoparticles can be strongly impacted by polymer degradation or changes in matrix properties, which can affect particle size, drug retention, and subsequent release kinetics during sterilization or prolonged storage. The translation of inorganic nanoparticles notably depends on reproducible control of their composition, surface chemistry, aggregation state, and persistence or clearance, which are all relevant to long-term ocular safety, even though such nanoparticles do not undergo lipid or polymer degradation in quite the same way. Hybrid nanoparticles consist of two or more components. Due to the incorporation of multiple components, hybrid nanoparticles inherit critical quality attributes from each of the components. However, variability is introduced at the interfaces. Thus, during scale-up, relative composition, architecture, drug distribution, and release behavior must be preserved across batches. Based on these differences, manufacturing controls, sterilization strategies, stability testing, and regulatory characterization will need to be class-specific rather than applied uniformly to all nanoformulations [20,23,60].

These class-specific considerations also explain why regulatory evaluation cannot focus on the active pharmaceutical ingredient alone. Manufacturing changes in complex nanomedicines may alter particle-size distribution, polydispersity, surface properties, drug loading or encapsulation state, impurities, aggregation, stability, or release behavior, making characterization of the carrier and the final formulation part of the therapeutic product rather than merely a manufacturing issue [29,53,54,60]. For regulatory characterization to occur, critical material and process attributes must be linked to the properties of the final product that are relevant to ocular exposure, pharmacodynamic performance, and safety. Local tolerance, persistence or degradation of carrier components, and, where relevant, systemic exposure should also be assessed for long-term ocular administration.

The incorporation of device-integrated and multiscale delivery systems gives rise to another manufacturing and regulatory interface, as the final therapeutic product is no longer just a nanoparticle suspension. The performance of contact lenses loaded with nanoparticles or micelles depends on both the characteristics of the nanocarrier and the performance of the lens as a drug reservoir. Changes in nanoparticle loading, incorporation into the lens matrix, release behavior, optical or physical properties, sterilization, or storage may alter the performance of the final product [55,56]. In the same way, iontophoretic delivery couples the nanoparticle formulation to an external physical delivery system, so performance depends on formulation characteristics together with device-dependent parameters and administration conditions [57]. Such systems thus require characterization and reproducibility of the entire formulation–device combination rather than extrapolation from the nanoparticle component alone. These interfaces are crucial for translation, particularly for chronic glaucoma therapy, where consistent manufacturing and repeatable administration are needed.

Manufacturing cost and patient acceptability add further translational constraints that cannot be inferred from physicochemical performance. Complex multistep production, specialized sterile processing, extensive analytical quality control, expensive raw materials, or device integration may reduce the practical advantage of an otherwise effective formulation. Patient acceptability is likewise route dependent: topical administration preserves familiarity and self-administration, whereas repeated injections, contact-lens handling, iontophoretic procedures, or other device-dependent strategies may introduce procedural, comfort, access, and healthcare-resource burdens. A clinically viable nanoengineered glaucoma therapy must therefore be not only effective, reproducible, and safe but also economically manufacturable and acceptable for repeated chronic use.

Future development should therefore move beyond demonstrating superiority to a conventional drug solution in a short experimental model. Studies should first establish which formulation parameter is responsible for the desired biological effect and define the relationship between drug loading, release, penetration, tissue exposure, and pharmacodynamic response. Comparators should use clinically relevant marketed formulations at clinically relevant doses and dosing regimens whenever possible; differences in dose, route, or administration frequency should be matched where appropriate or explicitly justified. Pharmacokinetic and pharmacodynamic endpoints should be prespecified and sufficiently standardized, and follow-up should correspond to the intended duration of clinical use rather than to an arbitrary experimental interval. Repeated-dose studies should evaluate safety for periods appropriate to the intended duration of use, and formulations that progress to humans require adequately powered trials assessing sustained IOP control together with ocular tolerability and clinically meaningful treatment burden. The limited human studies available to date [28,48] show that translation is possible, but they also define the current evidentiary gap. Closing this gap will require development programs that evaluate formulation performance, chronic route-specific safety, manufacturability, and clinically meaningful comparative benefit as linked requirements rather than as independent stages of development.

Ultimately, the value of nanotechnology in glaucoma does not arise from nanoscale dimensions themselves. The evidence reviewed here indicates that its principal advantage is the ability to engineer the relationship between drug loading, ocular retention, penetration, and release. At the same time, the studies also show that maximizing any one of these parameters does not guarantee a superior therapeutic outcome. The most credible candidates for clinical translation will therefore be formulations in which a specific limitation of existing therapy—such as poor solubility, insufficient penetration, rapid clearance, ocular-surface intolerance, or frequent administration—is addressed by a reproducible design whose pharmacological advantage is accompanied by appropriate long-term safety and manufacturability. At present, however, the available human evidence does not establish that nanoengineered glaucoma-delivery systems improve comparative clinical effectiveness, long-term safety, adherence, cost-effectiveness, or glaucoma-related disease outcomes over established therapy.

### 3.6. Emerging and Next-Generation Nanoenabled Strategies

Future formulations are more likely to extend beyond passive sustained release of established IOP-lowering drugs. A number of new strategies are emerging, which we can already start to cluster based on their therapeutic objective: some remain primarily focused on IOP control, while others aim at RGC targeting and/or preservation, neuroprotection, modulation of inflammatory or oxidative injury pathways, or sustained molecular intervention; the biological endpoints that each of these approaches will need are different, and we should not treat them as interchangeable. Newly developing platforms are being designed to provide biological responsiveness, cell-specific targeting, neuroprotection, sustained modulation of disease pathways, and sensing/treatment integration. However, these approaches differ markedly in maturity. Some already have proof of concept specific to glaucoma in experimental models, while others are mainly enabling technologies supported by broader ocular or nanopharmaceutical studies. They should thus not be interpreted as equivalent future therapeutic candidates.

To a large extent, conventional sustained-release nanocarriers are unable to solve the limitation posed by passive formulations, which release drugs according to predetermined physicochemical kinetics. In contrast, stimuli-responsive systems have the potential to alter drug release in accordance with the pathological microenvironment. There is now preliminary evidence for glaucoma. A research team led by Zhou developed a hypoxia- and reactive-oxygen-species (ROS)-responsive nano-prodrug in which hypoxia-sensitive and ROS-cleavable components promoted drug release under conditions associated with RGC injury and produced neuroprotective effects in experimental glaucoma models [61]. Because they connect drug availability to biological stress rather than time alone, such systems are conceptually appealing. However, responsiveness displayed in an experimental model does not prove that the triggering signal will be sufficiently specific, stable, or reproducible in chronic human glaucoma. Additionally, long-term safety, trigger thresholds, off-target release, and manufacturing reproducibility remain important translational questions.

Biomimetic carriers aim to imitate biological recognition or transport mechanisms, allowing for cell-specific delivery. Recently, researchers have been studying reconstituted high-density-lipoprotein nanoparticles as a biomimetic platform to target retinal ganglion cells and optic-nerve-head astrocytes through an uptake mechanism associated with apolipoprotein A-I/SR-B1. In vitro and ex vivo human-eye experiments have shown successful delivery to the retina and the retinal ganglion cell layer. However, therapeutic efficacy in glaucoma has not yet been established [62]. Thus, this methodology may assist in dealing with the limited cellular specificity of conventional nanocarriers. However, the current evidence is mostly limited to biodistribution and targeting, with no demonstrated neuroprotection or clinical efficacy. Extracellular vesicles (EVs), such as small EVs and exosome-enriched preparations, represent a related but biologically distinct strategy because they derive from intercellular communication pathways and can transport proteins, lipids, and regulatory RNAs. Research focusing specifically on glaucoma has shown RGC neuroprotection with stem-cell-derived EVs in experimental models, including the DBA/2J model, supporting a role beyond IOP lowering [63]. However, EV-based therapy remains preclinical. Unresolved concerns include cargo heterogeneity, source-cell dependence, biodistribution, persistence, dose definition, purification, storage, batch reproducibility, and scalable manufacture. Furthermore, repeated intraocular administration may in itself limit the theoretical reduction in treatment burden.

The delivery of nucleic acids broadens the therapeutic objective from the extended release of a conventional drug to sustained modulation of pathways relevant to the disease. The brinzolamide/miRNA-124 PEG–PSA nanoparticle system previously described in this review [58] offers glaucoma-specific proof of concept for combining intraocular pressure lowering with a molecular neuroprotective strategy, but remains experimental as an intravitreal approach. The idea could also be advanced through gene-based strategies aimed at altering aqueous-outflow pathways or RGC survival mechanisms for far longer than conventional pharmacological delivery.

One of the clearest examples of this evolution is CRISPR-associated strategies. In a model of MYOC-associated glaucoma, CRISPR-Cas9 editing reduced mutant myocilin-associated endoplasmic-reticulum stress, lowered IOP, and prevented glaucomatous damage. Furthermore, feasibility was also demonstrated in ex vivo human trabecular-meshwork tissue [64]. More recently, the trabecular meshwork was targeted using a cationic lipid–polymer lipoplex, which delivered Cas9 mRNA and guide RNA in a non-viral way to rescue ocular hypertension in a MYOC glaucoma mouse model [65]. Evidence from these studies is preclinical and addresses a genetically defined subset of disease; nevertheless, they provide meaningful glaucoma-specific mechanistic evidence. To be more widely applicable, durable delivery to target cells must be balanced against editing specificity, unintended genomic effects, inflammatory or immune responses, reversibility, manufacturing complexity, and the potential effects of long-lasting or irreversible biological modification.

Theranostic systems address another limitation of traditional sustained delivery. Treatment, which is usually given on a fixed schedule, does not provide ongoing information on the biological variable being treated. By integrating sensing and drug delivery, feedback-controlled therapy becomes possible. Preclinical evidence focused on glaucoma already exists for smart contact-lens platforms that couple continuous or responsive IOP sensing with on-demand drug delivery. A recent study involving an all-polymer theranostic contact lens integrated non-invasive IOP monitoring with pressure-gated release of timolol or brimonidine and demonstrated responsive IOP control in vitro, ex vivo, and in rabbit ocular-hypertension models [66]. Therefore, closed-loop systems are more than just prolonged delivery of drugs; these systems offer the prospect of adapting treatment to IOP changes as they occur. Nonetheless, the intricacy of these devices redirects the issue of translation toward various factors, including sensor accuracy, calibration, power or actuation requirements, comfort, device reliability and reproducibility, replacement interval, and demonstration that feedback-guided dosing improves clinically meaningful outcomes.

AI is an enabling technology, not a therapeutic platform, so it occupies a different position in the framework. Machine-learning approaches could help reduce empirical trial-and-error during the development of nanoformulations by modeling nonlinear relationships between composition, process parameters, particle size, polydispersity, drug loading, release, stability, pharmacokinetics, and toxicity. AI-based optimization of nanoformulations, release kinetics, and pharmacokinetic behavior has been proposed recently in the ocular-drug-delivery literature [67]. Nonetheless, there is currently no meaningful glaucoma-specific evidence that an AI-designed nanoparticle progresses more successfully from formulation optimization to therapeutic validation. AI must therefore be considered a potentially valuable formulation-development and decision-support tool rather than an established glaucoma-delivery technology. Its value for translation will rely on sufficiently large and standardized datasets, external validation, model interpretability, and prospective experimental confirmation of predicted formulations.

In sum, these emerging approaches should be judged by the unmet limitation they seek to overcome, not mere technological novelty. Stimuli-responsive systems may allow adaptive release triggered by pathological signals. Biomimetic carriers and EVs may improve cell-specific targeting and biological cargo delivery. Nucleic-acid and genome-editing strategies may enable sustained modification of disease pathways. Theranostic systems may couple continuous monitoring to feedback-controlled therapy. AI may accelerate formulation optimization and selection. Among these approaches, preclinical evidence in glaucoma already exists for responsive nanotherapeutics, EV-mediated neuroprotection, targeted gene or genome editing, and closed-loop theranostic devices, whereas clinical evidence remains absent. The main question for future development is not whether these technologies are more sophisticated than existing nanocarriers, but whether they can solve a clinically relevant problem, such as RGC-specific neuroprotection, durable gene modulation, or adaptive drug release, while maintaining the safety, reproducibility, manufacturability, route acceptability, and clinical benefit needed for chronic glaucoma management.

### 3.7. Comparative Characteristics of Major Nanoengineered Platforms

The leading nanoengineered platforms researched for glaucoma vary fundamentally in their architecture, drug-loading mechanism, release control, interaction with ocular barriers, manufacturing needs, and level of translational development. Making a direct comparison based only on particle size or duration of IOP lowering is therefore inappropriate. Table 5 compares polymeric nanoparticles, liposomes, nanomicelles, niosomes, dendrimers, nanocrystals, stimuli-responsive nanoparticles, and nanoparticle-loaded hydrogels. The objective of this comparison is to assess the specific strengths and weaknesses of each platform rather than to rank their efficacy against one another, because substantial differences exist in the underlying studies in terms of drug, administration route, dose, comparator, experimental model, and follow-up.

Dendrimers must be considered separately since their highly branched architecture and multiple terminal functional groups allow extensive surface modification and covalent or non-covalent association with therapeutic molecules. In preclinical investigations for glaucoma, DenTimol, a third-generation PAMAM dendrimer conjugated to a timolol precursor, was devised by Lancina et al. About 8% of the dendrimeric drug permeated excised rabbit cornea within 4 h; topical application produced a ~7.3-mmHg fall in IOP in normotensive Brown Norway rats; and daily application for 7 days did not produce any histologically detectable ocular toxicity [68]. More recently, nano-in-nano dendrimer hydrogel particles of approximately 200 nm were used to deliver brimonidine and timolol. The particles enhanced corneal permeability nearly 17-fold relative to plain drug solution. Further, these particles provided prolonged near-zero-order release and also showed IOP-lowering activity in Brown Norway rats and experimental glaucoma mice [69]. These results show glaucoma-specific proof of concept in animals but not human efficacy or long-term safety.

Nanoparticle-loaded hydrogels represent a related but structurally distinct multiscale strategy in which the nanoscale carrier and surrounding gel matrix perform complementary functions. The nanoparticle is useful in regulating drug loading, release, or tissue interaction, while the hydrogel can enhance precorneal residence and generate an additional sustained-release depot. Yang and coworkers used a PAMAM dendrimer hydrogel in combination with PLGA nanoparticles for topical co-delivery of brimonidine and timolol. The formulation sustained ocular drug exposure for up to one week, and a single topical administration produced effective IOP reduction for four days in rabbits [70]. Notably, the therapeutic performance of such compositions is representative of the entire nanoparticle–hydrogel formulation and cannot simply be ascribed to the nanoparticle component.

**Table 5:** Comparative characteristics of major nanoengineered platforms investigated for glaucoma drug delivery.

Platform	Representative Drug Loading/Encapsulation	Predominant Release Mechanism(s)	Main Administration Route(s)	Principal Advantages	Principal Limitations	Demonstrated Glaucoma Applications/Evidence Maturity
Polymeric nanoparticles	Formulation-dependent; e.g., timolol/chitosan EE 75.34% [40]; dorzolamide PCL/chitosan EE 72.48% [45]	Diffusion combined with polymer degradation and/or erosion; initial burst may occur	Topical; subconjunctival; intracameral; intravitreal; iontophoresis-assisted	Tunable degradation and release; surface functionalization; mucoadhesion; potential for sustained and combination delivery	Burst release; polymer-dependent degradation and tissue responses; degradation products; sterilization and scale-up sensitivity; uncertain long-term disposition	Extensive preclinical formulation, transport, PK and animal PD evidence [26,40,41,42,43,44,45,46,57,58]; no established human clinical efficacy
Liposomes	LAT-HA-LIP EE ≥ 99.46% [30]; brinzolamide liposomes EE 95.41% and DL 4.00% [34]	Drug diffusion/partitioning through the lipid bilayer; leakage may contribute	Topical; subconjunctival; suprachoroidal	Can accommodate hydrophilic and lipophilic drugs; adaptable surface composition; high encapsulation achievable; sustained delivery	Drug leakage; lipid oxidation and phase changes; aggregation; storage instability; sterilization sensitivity	Extensive preclinical evidence [30,31,34]; first-in-human subconjunctival latanoprost feasibility, *n* = 6 [28]; suprachoroidal preclinical latanoprost evidence [71]; no established comparative clinical efficacy
Nanomicelles	Drug loading depends on drug–core interactions and is formulation-specific	Micellar diffusion/dissociation; an external device reservoir may additionally regulate release	Topical/device-integrated contact lens	Solubilization of poorly water-soluble drugs; small dimensions; tunable surface chemistry; compatibility with larger delivery devices	Stability may depend on dilution and formulation conditions; drug-loading capacity is drug-dependent; nanocarrier and device effects can be difficult to separate	Mainly preclinical and device-integrated evidence [52,53,54,55]; no established clinical efficacy
Niosomes	Brimonidine EE 79.23% [33]; acetazolamide EE up to 32.21% [39]	Diffusion/partitioning through the surfactant bilayer	Mainly topical	Flexible surfactant composition; tunable entrapment and release; potential for prolonged topical delivery	Aggregation, fusion and leakage; strong dependence on surfactant type, cholesterol ratio and preparation method	Preclinical formulation and animal PD evidence with brimonidine and acetazolamide [33,39]; no human evidence
Dendrimers	Multivalent covalent or non-covalent drug association; conventional EE may not always be applicable [68,69]	Linker cleavage and/or diffusion/network-controlled release depending on architecture	Mainly topical	Extensive surface functionalization; multivalent drug association; potential mucoadhesion and multidrug delivery	Surface-charge-dependent cytotoxicity; synthetic and characterization complexity; uncertain long-term ocular fate and scalable manufacture	Glaucoma-specific preclinical evidence with DenTimol and dendrimer gel particles [68,69]; no human evidence
Nanocrystals	Conventional encapsulation not applicable because the nanoscale phase consists predominantly of active drug	Dissolution-controlled	Topical	Very high drug fraction; enhanced dissolution of poorly soluble agents; limited carrier burden	Aggregation and physical instability; stabilizer dependence; dissolved/free-drug fraction remains important; sustained release is not intrinsic	Preclinical brinzolamide evidence with rapid dissolution and experimental IOP lowering [47]
Stimuli-responsive nanoparticles	Architecture-specific; conventional EE may not be applicable	Trigger-dependent activation, cleavage or release, including hypoxia/ROS-responsive mechanisms	Experimental ocular/intraocular administration	Potential adaptive release linked to pathological signals; may couple delivery with biologically targeted or neuroprotective strategies	Trigger threshold and specificity; biological variability; off-target activation; manufacturing complexity; uncertain long-term safety	Early glaucoma-specific preclinical proof of concept with a hypoxia/ROS-responsive nanoprodrug [61]; no human evidence
Nanoparticle-loaded hydrogels	Drug may be incorporated in the nanoparticle, hydrogel, or both [69,70]	Nanoparticle diffusion/release combined with hydrogel swelling, degradation and/or erosion	Mainly topical/in situ-gelling systems	Combines nanoscale drug control with increased precorneal residence or depot formation; potential multidrug delivery	Pharmacological effects cannot be attributed to nanoparticles alone; rheology, gelation, dose reproducibility, sterilization, storage and manufacturing require control	Preclinical glaucoma evidence with dendrimer-gel and dendrimer-hydrogel/PLGA nanoparticle systems [69,70]; no human evidence

Note: Drug-loading and encapsulation values are representative study-specific examples and should not be interpreted as standardized cross-platform performance measures. Release mechanisms may coexist within individual formulations. The comparison summarizes platform architecture, principal potential advantages and limitations, administration routes, and the maturity of glaucoma-specific evidence rather than ranking therapeutic efficacy. Hybrid and multiscale systems may span more than one category, and their pharmacological effects should be interpreted at the level of the complete formulation.

Physicochemical characterization should be read as a global assessment of formulation performance rather than as a set of independent descriptors. The dispersion behavior, ocular residence, tissue interaction, and clearance of particles depend on their size, while the polydispersity index indicates the heterogeneity of the particle-size distribution. Colloidal stability, mucoadhesion, interaction with membranes, and association with tissues are influenced by zeta potential and surface chemistry. Particle shape could also alter surface area, drug–carrier interactions, and release behavior. Drug loading and encapsulation efficiency refer to two different things. Drug loading is the amount of drug relative to the carrier or formulation, whereas encapsulation efficiency is the fraction of the drug added initially that is retained in or associated with the carrier. We must also monitor colloidal stability over time, as aggregation, growth of particles, leakage of drugs, or changes in surface properties could alter their ocular disposition despite no change in total drug concentration. The release kinetics of an ocular formulation integrate many of these attributes by determining the concentration–time profile available for ocular absorption and pharmacological activity [20,23,29,53,54,60].

The mechanism of drug release from nanoengineered ocular systems may involve various processes that should not be considered interchangeable. In diffusion-controlled systems, the drug moves through the carrier matrix, membrane, or surrounding vehicle according to a concentration gradient. In degradation-controlled systems, the breakdown of the carrier, whether chemical or enzymatic, causes progressive alteration of the mobility of the drug. Erosion-controlled release, on the other hand, depends on the loss of material from either the surface or the bulk of the carrier. Systems containing a hydrogel may also exhibit swelling-controlled release as water uptake expands the polymeric network. It is possible to have these mechanisms in one formulation. Stimuli-responsive systems operate via a distinct mechanism whereby drug release or disassembly of the carrier changes in response to a biological signal. The hypoxia/ROS-responsive system described above ref. [61] is one example.

Initial burst release is another formulation-dependent phenomenon of significance. Drug located near the particle surface or present as a free or weakly associated fraction may be rapidly released after administration, allowing a slower, sustained release phase to follow. An initial release burst was observed with timolol-loaded gelatin nanoparticles, for example [41]; biphasic release was observed with dorzolamide-loaded chitosan-coated PCL nanoparticles [45]. Although a limited burst can provide rapid therapeutic onset, an excessive burst can diminish the fraction of drug available for prolonged release and may elevate early local exposure and toxicity. Sustained-delivery performance should thus be interpreted on the basis of the complete release profile rather than on the duration of the terminal release phase alone.

There is a trade-off between ocular retention and biocompatibility in relation to surface charge. Cationic surface characteristics can promote electrostatic interaction and mucoadhesion due to the presence of negatively charged components on the ocular surface and within the mucin layer, as exploited in chitosan-containing and various other positively charged formulations [45,51]. But it is not necessarily beneficial to increase positive surface charge. Stronger interactions with cellular membranes may increase membrane disruption, cellular uptake, inflammatory responses, or cytotoxicity. Zeta potential should therefore be optimized together with carrier composition, dose, exposure duration, and tissue-specific tolerability rather than maximized as an independent formulation target [20,23,60].

These route-specific differences are summarized comparatively in Table 6.

The literature on nanoengineered glaucoma covers many established drug classes. Prostaglandin analogs include latanoprost and travoprost formulations [28,30,31,36,37,38,55,57]; β-blockers mainly include timolol formulations [25,35,40,41,42,55,56,59]; carbonic-anhydrase inhibitors include acetazolamide, brinzolamide, and dorzolamide formulations [34,35,39,45,46,47,48,49,58]; and α2-adrenergic agonists include brimonidine formulations [32,33,43,44]. Pilocarpine-based systems are also cholinergic outflow-modifying therapies [26,27,50,51]. The contemporary IOP-lowering class of Rho-kinase inhibitors, including netarsudil and ripasudil, principally acts through modification of conventional outflow [72]. Despite this, the primary studies in the core formulation–performance synthesis did not feature any nanoparticle-specific delivery of clinically established ROCK inhibitors. Owing to this, an evidence gap exists, and nanoparticle efficacy should not be extrapolated from conventional ROCK-inhibitor therapy.

The models discussed in this literature examine different biological questions and should not be considered interchangeable. The formulation–performance evidence involves normotensive animals and glaucoma or ocular-hypertension models created using different methodologies, whereas neuroprotective studies additionally use injury models that isolate individual components of RGC degeneration. The brinzolamide/miRNA-124 study examined IOP-lowering activity in a microbead-induced ocular-hypertension model but assessed RGC survival separately after optic-nerve crush [58]. More recent studies also include genetic glaucoma models such as DBA/2J mice and experiments that induce retinal or optic-nerve injury [61,63,64,65]. The various models represent different aspects of glaucomatous disease and should not be interpreted as equivalent models of chronic human glaucoma.

Differences between species further limit direct translation of nanoparticle performance. Ocular dimensions, tear dynamics, corneal and conjunctival anatomy, aqueous- and vitreous-humor volumes, outflow anatomy, tissue permeability, and drug clearance differ among mice, rats, rabbits, and humans. As a result, ocular residence, tissue exposure, and the duration of IOP lowering seen in experimental animals cannot be directly translated into a human dosing interval. Preclinical research involving animal species is very useful for pharmacokinetic, pharmacodynamic, and safety proof of concept. However, ultimately, clinically meaningful durability and dosing require confirmation in studies conducted in humans [17,20,23].

It is necessary to distinguish between localization of the intact nanocarrier and localization of the released drug in ocular disposition. The reviewed studies show prolonged ocular-surface association in selected systems [38], increased transcorneal or conjunctival–scleral drug transport [31,34,45], increased exposure in ciliary-body or aqueous-humor compartments [27,37,57,59], and experimental delivery toward retinal or RGC targets [58,62,63]. New gene-delivery systems have also targeted the trabecular meshwork [65]. Yet, several studies quantify drug concentrations rather than the biodistribution of intact nanoparticles. Therefore, enhanced drug exposure does not necessarily indicate that intact nanoparticles crossed the same anatomical barriers. Systemic absorption, nanoparticle clearance, and long-term carrier fate remain poorly defined.

## 4. Materials and Methods

This narrative review was carried out using a search strategy to identify literature concerning nanoparticle-mediated and nanoengineered drug-delivery approaches for glaucoma and ocular hypertension. PubMed/MEDLINE was searched without a lower date restriction, with the final search performed on 26 August 2026. Terms related to glaucoma, ocular hypertension, intraocular pressure (IOP), and ocular drug delivery were combined with terms related to nanoscale carriers, formulation components, delivery platforms, and individual IOP-lowering agents.

**Figure 3:**
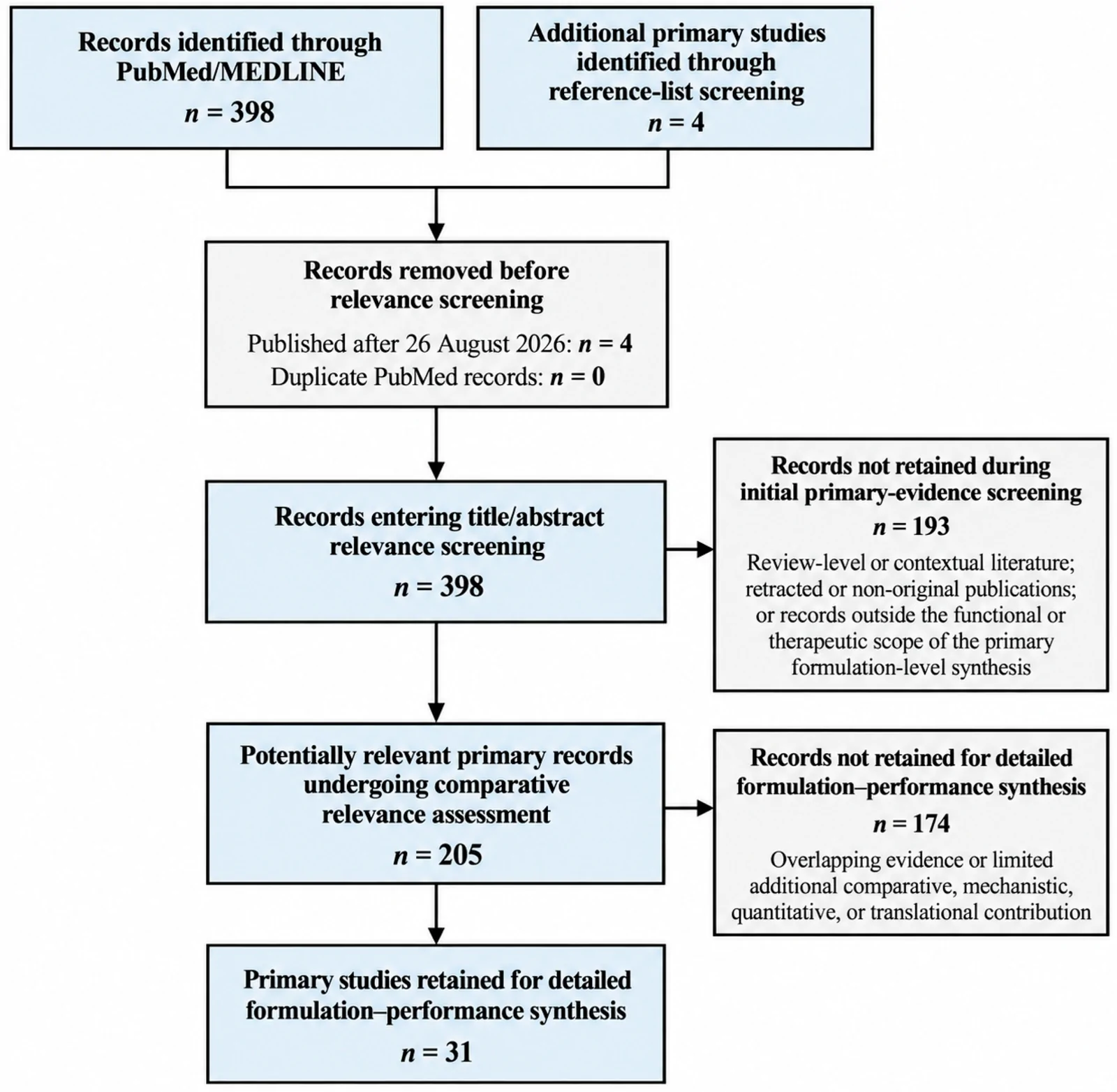
Flow diagram of literature identification and study selection for the detailed formulation–performance synthesis.

The search was conducted using combinations of the terms “glaucoma”, “ocular hypertension”, “intraocular pressure”, “ocular drug delivery”, “nanoparticle”, “nanocarrier”, “nanotechnology”, “liposome”, “solid lipid nanoparticle”, “nanostructured lipid carrier”, “niosome”, “polymeric nanoparticle”, “PLGA”, “PLA”, “polycaprolactone”, “chitosan”, “gelatin nanoparticle”, “nanomicelle”, “nanosuspension”, “nanocrystal”, “cyclodextrin nanoparticle”, “nanoemulsion”, “hybrid nanoparticle”, and “contact lens”, in combination with established or investigational IOP-lowering agents including latanoprost, travoprost, timolol, brimonidine, brinzolamide, dorzolamide, acetazolamide, and pilocarpine. To provide a reproducible record of the literature search, the PubMed/MEDLINE search was rerun on 15 September 2026 while retaining 26 August 2026 as the upper publication-date cutoff. The complete Boolean search strategy used for this reproducibility search was: ((“glaucoma”[Title/Abstract] OR “ocular hypertension”[Title/Abstract] OR “intraocular pressure”[Title/Abstract]) AND (“ocular drug delivery”[Title/Abstract] OR nanoparticle*[Title/Abstract] OR nanocarrier*[Title/Abstract] OR nanotechnolog*[Title/Abstract] OR liposome*[Title/Abstract] OR “solid lipid nanoparticle”[Title/Abstract] OR “solid lipid nanoparticles”[Title/Abstract] OR “nanostructured lipid carrier”[Title/Abstract] OR “nanostructured lipid carriers”[Title/Abstract] OR niosome*[Title/Abstract] OR “polymeric nanoparticle”[Title/Abstract] OR “polymeric nanoparticles”[Title/Abstract] OR PLGA[Title/Abstract] OR PLA[Title/Abstract] OR polycaprolactone[Title/Abstract] OR chitosan[Title/Abstract] OR “gelatin nanoparticle”[Title/Abstract] OR “gelatin nanoparticles”[Title/Abstract] OR nanomicelle*[Title/Abstract] OR nanosuspension*[Title/Abstract] OR nanocrystal*[Title/Abstract] OR “cyclodextrin nanoparticle”[Title/Abstract] OR “cyclodextrin nanoparticles”[Title/Abstract] OR nanoemulsion*[Title/Abstract] OR “hybrid nanoparticle”[Title/Abstract] OR “hybrid nanoparticles”[Title/Abstract] OR “contact lens”[Title/Abstract] OR “contact lenses”[Title/Abstract] OR iontophoresis[Title/Abstract] OR iontophoretic[Title/Abstract] OR “nanostructured microparticle”[Title/Abstract] OR “nanostructured microparticles”[Title/Abstract] OR mucoadhesive[Title/Abstract]) AND (latanoprost[Title/Abstract] OR travoprost[Title/Abstract] OR timolol[Title/Abstract] OR brimonidine[Title/Abstract] OR brinzolamide[Title/Abstract] OR dorzolamide[Title/Abstract] OR acetazolamide[Title/Abstract] OR pilocarpine[Title/Abstract] OR “IOP-lowering”[Title/Abstract] OR “intraocular pressure lowering”[Title/Abstract])). No publication-type restriction was applied at the database-search stage because in vitro, ex vivo, animal, human, and review-level evidence was potentially relevant to the objectives of this narrative review. No language restriction was applied during the database search. Records with a publication date later than 26 August 2026 were removed before title and abstract screening in order to preserve the prespecified publication cutoff. Recent reviews of ocular nanotechnology and glaucoma drug-delivery systems, as well as the reference lists of eligible publications, were screened to identify relevant primary studies. Publications retrieved through the database search and reference-list screening were assessed for relevance to the objectives of the review, and potentially eligible studies were subsequently evaluated according to the inclusion and exclusion criteria described below. During revision, targeted supplementary PubMed searches were additionally performed to ensure adequate platform-level coverage of delivery classes not explicitly represented in the original Boolean strategy. These searches combined ‘glaucoma’, ‘ocular hypertension’, ‘intraocular pressure’, or ‘ocular drug delivery’ with ‘dendrimer’, ‘dendrimeric’, ‘hydrogel’, ‘nanogel’, ‘nanoparticle-loaded hydrogel’, ‘in situ gel’, ‘stimuli-responsive’, ‘stimulus-responsive’, ‘ROS-responsive’, or ‘hypoxia-responsive’. The same publication-date cutoff of 26 August 2026 was retained. These supplementary searches were used to support the qualitative platform-level comparison and did not alter the predefined detailed formulation–performance evidence subset summarized in Figure 3 and Table 4.

Studies were considered eligible for the primary evidence synthesis when they directly evaluated nanoengineered delivery of an IOP-lowering or glaucoma-directed therapeutic agent. Systematic reviews, meta-analyses, and recent narrative reviews were used primarily to establish the pharmacological and translational context and to identify relevant primary studies. If older papers provided foundational support for a delivery strategy, they were retained. In vitro studies were considered when they provided information on particle characteristics, drug loading and release, stability, and/or cytotoxicity. Ex vivo studies were considered when they evaluated corneal or ocular-tissue transport. Animal studies were evaluated when they considered pharmacokinetics, tissue distribution, tolerability, and/or IOP reduction or other glaucoma-relevant pharmacodynamic outcomes. Human trial data were assessed separately as they were more relevant to translation.

For this review, we operationally defined nanoparticle-mediated or nanoengineered delivery as a glaucoma-directed drug-delivery strategy in which a nanoscale drug phase, nanoparticle, nanocarrier, or nanoscale structural component makes a functional contribution to drug loading, solubilization or dissolution, ocular retention, tissue penetration, controlled release, or ocular exposure. Consequently, functional contribution served as the basis for eligibility rather than solely the dimensions of the final dosage form. For analytical purposes, the included systems were classified according to the functional role of the nanoscale component rather than solely according to material composition. Five operational categories were distinguished: (1) carrier-based nanosystems, in which the therapeutic agent is incorporated within or associated with a nanoscale carrier or dispersed phase, including lipid- and polymer-based nanoparticles, liposomes, niosomes, nanomicelles, dendrimer-based carriers, nanoemulsions, cyclodextrin-based nanoparticles, liquid-crystalline nanostructures, and related hybrid carriers; (2) drug nanocrystals or nanosuspensions, in which the nanoscale phase consists predominantly of the active drug and nanosizing primarily modifies dissolution or the available drug fraction; (3) nanoscale surface or permeability modifiers, in which nanoparticles are not the principal drug reservoir but modify tissue interaction, permeability, or local drug disposition; (4) multiscale nano-enabled formulations, in which a larger dosage form contains functionally relevant nanoscale architecture, as in nanostructured microparticles or nanoparticle-loaded hydrogel and in situ-gelling composite systems; and (5) nanoparticle- or nanocarrier-integrated delivery devices, in which a nanoscale formulation is incorporated into or delivered through a larger device or physical delivery system, including nanoparticle- or micelle-loaded contact lenses and nanoparticle formulations administered by iontophoresis. Stimuli-responsive behavior was considered a functional design characteristic that can occur across these structural categories rather than a mutually exclusive material class. These categories describe the predominant delivery role of the nanoscale component and should not be interpreted as equivalent material classes. In particular, therapeutic performance in multiscale and device-integrated systems may reflect the combined effects of the nanoscale component, external vehicle, administration route, and delivery device rather than the nanoparticle alone. Delivery systems considered outside the main evidence synthesis were those without a functionally relevant nanoscale component, including conventional contact lenses, microparticles, iontophoretic approaches, or other delivery devices. As such, we defined these platforms as distinct nano-enabled delivery architectures, not equivalent nanoparticle classes. Records were first screened by title and abstract against these eligibility criteria. Full-text articles were subsequently assessed when eligibility could not be established from the title and abstract alone. Duplicate records were identified using PMID or DOI when available and, when necessary, by comparison of title, authors, publication year, and journal. During eligibility assessment, reasons for exclusion were recorded according to the predefined eligibility criteria, including lack of relevance to glaucoma or ocular hypertension, absence of an IOP-lowering or glaucoma-directed therapeutic strategy, absence of a functionally relevant nanoengineered component, or absence of original formulation, pharmacokinetic, pharmacodynamic, or safety data relevant to the primary evidence synthesis. Given the narrative and comparative scope of the review, the database search was used to identify the broader evidence base rather than to impose exhaustive systematic inclusion of every retrieved primary study. For the detailed formulation–performance synthesis, priority was given to primary studies providing quantitative evidence linking formulation architecture or physicochemical characteristics to ocular exposure, pharmacokinetic or pharmacodynamic performance, tolerability, or human translation. When multiple studies addressed closely similar drug–platform combinations, studies providing the most complete comparative, mechanistic, or translational information were prioritized. Study selection and data extraction were performed by M.C. and subsequently verified by M.Z.; discrepancies regarding eligibility or extracted information were resolved by discussion and consensus. The literature-identification and study-selection process for the detailed formulation–performance synthesis is summarized in Figure 3.

The reproducibility search identified 398 records in PubMed/MEDLINE. Four records published after the predefined cutoff of 26 August 2026 were removed before relevance screening, and no duplicate PubMed records were identified. Four additional primary studies were identified through reference-list screening, resulting in 398 records entering relevance screening. Of these, 193 were not retained during the initial primary-evidence screening because they represented review-level or contextual literature, retracted or non-original publications, or did not meet the functional and therapeutic scope of the primary formulation-level synthesis. The remaining 205 potentially relevant primary records underwent comparative relevance assessment. Of these, 174 were not retained for the detailed formulation–performance synthesis because they provided overlapping evidence or limited additional comparative, mechanistic, quantitative, or translational information relative to the studies prioritized for detailed analysis. Thirty-one primary studies were therefore retained for the detailed formulation–performance synthesis.

Researchers did not consider retracted studies. Whenever possible, bibliographic information and publication status were checked against PubMed or the relevant journal record. Since the goal was a narrative and mechanistic synthesis, and not the estimation of a pooled treatment effect, no original meta-analysis was performed. Formal study-quality appraisal was considered but was not undertaken because the evidence base comprised fundamentally different study types, including formulation-only experiments, ex vivo ocular-tissue studies, animal models of ocular hypertension or optic-nerve injury, and human clinical studies, for which no single risk-of-bias instrument is applicable across all designs. Applying separate design-specific instruments would generate methodological judgments that are not directly comparable across these evidence classes and would not provide a meaningful common quality hierarchy for this narrative synthesis. We therefore used a structured descriptive appraisal at the study level, recording the experimental model, sample size where reported, comparator, dosing regimen, follow-up duration, tolerability findings, and principal methodological limitations. These characteristics and study-specific limitations are reported in Table 4, while the inferential limits associated with each level of evidence are summarized in Table 2. This approach should not be interpreted as a formal risk-of-bias assessment, and the resulting limitations in confidence across the evidence base are explicitly considered in the Section 3.

The findings extracted were categorized by delivery strategy and translational relevance. Close attention was paid to the composition and architecture of the carrier, particle characteristics, drug loading and release, route of administration, corneal or ocular penetration, pharmacokinetics, duration of pharmacodynamic action, ocular tolerability, and level of evidence. Physicochemical and ex vivo improvements were distinguished from demonstrated in vivo pharmacodynamic effects, while human findings were considered separately from preclinical evidence. For each primary study, additional structured information was recorded where reported, including publication year, drug, carrier composition and formulation architecture, particle size and polydispersity, administration route, experimental model and sample size, comparator, dosing regimen, follow-up duration, principal pharmacokinetic or ocular-exposure outcomes, pharmacodynamic outcomes, tolerability findings, and key methodological limitations. All major study-specific quantitative statements in the narrative synthesis and summary tables were cross-checked against the corresponding primary publication wherever available; secondary reviews were retained for contextual, mechanistic, or translational background but were not used as the sole source for study-specific numerical claims.

## 5. Conclusions

Drug-delivery systems manufactured at the nanoscale are promising experimental systems that can help overcome specific pharmacokinetic and treatment-burden limitations of traditional glaucoma therapy. The predominantly preclinical literature reports that these systems can alter drug solubility, ocular retention, penetration, release, tissue exposure, and, in many animal models, the duration of IOP reduction. Nonetheless, the available human evidence relies on small early studies, which are insufficient to establish comparative clinical effectiveness, long-term safety, adherence benefit, cost-effectiveness, or meaningful effects on glaucoma progression. For clinical translation, reproducible manufacturing, route-specific chronic safety, clinically relevant comparators and dosing regimens, patient-centered outcomes, and adequately powered human trials demonstrating a meaningful advantage over established therapy will be required. Nanoengineered strategies designed to offer neuroprotective or disease-modifying effects represent a less mature and distinct translational pathway, which will require direct evidence of retinal ganglion cell and optic-nerve preservation beyond effects attributable to IOP lowering.

## Figures and Tables

**Table 1:** Comparative clinical and translational framework of representative nanoengineered glaucoma drug-delivery systems.

Platform/Ref.	Nanoscale Component	Final Dosage Form and Complete Delivery Route	Experimental Model/Evidence Label	Comparator	Dose/Regimen; Follow-Up	Principal PK/Transport Endpoint	Principal PD/IOP Endpoint	Major Safety Finding	Effect Attribution	Major Translational Limitation
Surface-modified SLNs/timolol [25]	Phospholipid surface-modified SLNs	Nanoparticle dispersion; ex vivo corneal exposure	Bioengineered human cornea; Ex vivo transport evidence	Free timolol; unmodified SLNs	Dose/follow-up NR in extracted data	Papp 1.33–2.31 × 10^−5^ cm/s vs. 4.27 × 10^−5^ cm/s for free timolol; more sustained permeation	Not assessed	NR	Predominantly nanoformulation effect on release/permeation; no intact-eye PD evidence	No in vivo IOP or human efficacy evidence
Hollow PLA nanoparticles/pilocarpine [26]	Hollow PLA NPs with tunable shell thickness	Intracameral nanoparticle injection	Glaucomatous rabbits; Animal pharmacodynamic evidence	Different shell architectures	Single intracameral dose; >56 d	~40-nm shell: EE 86%; therapeutic drug levels >56 d	Ocular-hypertension control >56 d	Corneal endothelial, retinal, and optic-nerve structure/function preserved in reported assessments	NP architecture contributes to release, but prolonged performance also depends on invasive intracameral administration	Invasive route; repeat-administration and long-term intraocular safety unknown
Liposomal latanoprost [28]	Latanoprost liposomes	Liposomal depot; subconjunctival injection	Humans with OHT/POAG, *n* = 6; Human feasibility evidence	None	Single injection; 3 mo	Not directly assessed	IOP 27.55 ± 3.25 → 14.52 ± 3.31 mmHg at 1 h; ≥20% reduction significant through 3 mo	No VA loss; transient discomfort; one subject with pre-existing OSD had discomfort/blur	Whole-system effect: liposomal depot + subconjunctival administration; clinical contribution of the liposome cannot be isolated from the route	*n* = 6; open-label, non-comparative, non-randomized; repeat-injection and long-term safety unknown
LAT-HA-LIP/latanoprost [30,31]	Latanoprost liposomes within HA/osmoprotectant formulation	Topical eye-drop formulation	Cells, ex vivo porcine tissues, rabbits; Ex vivo transport + animal pharmacodynamic evidence	Marketed latanoprost; saline for selected outcomes	0.005% LAT; single-dose effect to 48 h; repeated daily dosing 15 d; safety monitoring 25 d	Greater LAT-acid tissue penetration; study-defined IOP-effect-AUC-derived relative ocular bioavailability 2.97-fold [30]	Single-dose effect 48 h vs. 24 h comparator; repeated-dose IOP reduction 2.51–3.88 mmHg on days 5–15	Ocular-surface parameters broadly physiological; minor hyperemia reported	Whole-formulation effect: liposomes + HA-containing vehicle + osmoprotectants	Human efficacy absent; chronic safety/efficacy not established
Lipid–DNA nanoparticles/travoprost [38]	Self-assembled amphiphilic DNA NPs + aptamer	Topical nanoparticle formulation	Porcine cornea + rats; Ex vivo transport + animal ocular-exposure + animal pharmacodynamic evidence	Free travoprost/free aptamer	Single topical dose; exposure assessed to 4 h; PD at 12 h	~4-fold greater ocular delivery at 4 h	Responders 50% vs. 33%; IOP response difference not significant, *p* = 0.1	NR	NP architecture clearly increased ocular delivery, but superior PD efficacy was not established	Increased exposure cannot be assumed to confer therapeutic superiority
Bioadhesive polymeric nanoparticles/brimonidine [44]	Chitosan/alginate nanoparticles	NP-containing topical eye-drop/gel vehicles	Animal model; Animal pharmacodynamic evidence	Alphagan^®^ P	Topical formulations; follow-up to 25.2 h	No direct PK endpoint	Tmax 5.6–7.2 vs. 2.2–2.4 h; duration 17.2–25.2 vs. 7.0–7.4 h; maximal IOP reduction not significantly greater	Cell viability 88.78–97.43%	Combined NP + external-vehicle effect; bioadhesion and vehicle both contribute	Human validation absent; NP-specific contribution cannot be completely isolated
Chitosan-coated PCL nanoparticles/dorzolamide [45]	Chitosan-decorated PCL NPs	Topical formulation evaluated ex vivo	Ex vivo ocular tissue; Ex vivo transport evidence	Dorzolamide solution	Release assessed to 12 h	Permeation 195.23 vs. 94.23 μg/cm^2^; mucoadhesion increased ~3.7-fold	Not assessed	Favorable histopathology/HET-CAM findings	NP/coating effect demonstrated for mucoadhesion and transport	No in vivo IOP experiment; evidence is mechanistic/transport rather than therapeutic
Brinzolamide nanocrystals [47]	HPMC-stabilized drug nanocrystals	Topical nanosuspension	Laser-induced OHT rats; Animal pharmacodynamic evidence	Azopt^®^; unmilled drug	Single 100-μg topical dose; acute follow-up	~100% dissolution within 1 min, but dissolution alone did not explain PD differences	Best formulation: 71.4 ± 5.0% IOP reduction at 60 min vs. 49.6 ± 16.5% with Azopt^®^	Mild/no toxicity after physiologically relevant dilution	Drug nanosizing contributes, but dissolved/free-drug fraction and formulation conditions also influence response	Acute animal evidence only; no human validation
γ-Cyclodextrin/dorzolamide nanoparticles [48]	γ-Cyclodextrin-based nanoparticles	Topical eye drops	Humans with IOP >18 mmHg, *n* = 17; Human feasibility evidence	Trusopt^®^	One 3% NP dose vs. three Trusopt administrations; 24 h	Not directly assessed	4-h IOP reduction 18% vs. 14%, *p* = 0.97; no significant between-treatment difference at 24 h	Burning score 12 ± 15 vs. 37 ± 30; VA/hyperemia similar	Whole topical nanoformulation effect; short-term human comparison	Small, 24-h study; equivalence/non-inferiority not established
Nanostructured mucoadhesive microparticles/dorzolamide [49]	PLGA/PEG nanostructured surface	Microscale mucoadhesive particles in rapidly dissolving PVA tablet; topical	Preclinical ocular model; Animal pharmacodynamic evidence	Trusopt^®^; intermediate formulations	Same dorzolamide dose; 10 h	No direct PK endpoint	Max IOP reduction 24.40 ± 2.31% vs. 17.96 ± 1.62%; duration 10 vs. 5 h; IOP-effect AUC 115.85 ± 12.30 vs. 51.46 ± 7.74	NR	Whole multiscale-system effect: nanosurface + mucoadhesion + microparticle architecture + tablet dosage form	Final dosage form is microscale; no human validation
Micelle-loaded contact lens/timolol + latanoprost [55]	mPEG–PLA drug-loaded micelles	Micelle-loaded contact lens	Elevated-IOP rabbits; Animal pharmacokinetic + animal pharmacodynamic evidence	Conventional eye drops	Lens: 100 μg timolol + 1 μg LAT; comparator: 250 μg + 2.5 μg; >168 h	Tear-fluid MRT +79.6-/122.2-fold; study-defined BA +2.2-/7.3-fold	IOP reduction >168 h; relative pharmacological availability 9.8-fold	Experimental ocular safety assessments favorable	Whole-system effect: micelles + contact-lens reservoir; not attributable to micelles alone	No chronic human wear data; device tolerability and manufacturing requirements
PLGA nanoparticles + iontophoresis/latanoprost [57]	Latanoprost-loaded PLGA NPs	NP-loaded PVA tablet + transdermal ocular iontophoresis	Rabbits; Animal pharmacokinetic + animal pharmacodynamic evidence	Xalatan^®^	~170–187 μg LAT in NP tablet; iontophoresis 4 mA × 30 min; Xalatan drop 0.175 μg; exposure to 12 d	Aqueous-humor drug AUC > 23-fold vs. Xalatan^®^	IOP-effect AUC > 16-fold; effect >7 d	No significant damage in reported acute assessments	Whole-system effect: NPs + iontophoresis + administration conditions; not an isolated NP effect	Device/procedure dependence; strong dose imbalance; AUC ratios not dose-normalized; repeated-use safety unknown
Mg(OH)_2_ nanoparticles + timolol [59]	Mg(OH)_2_ NPs acting as permeability modifiers	Topical co-instillation with dissolved timolol	Experimental ocular models; Animal pharmacokinetic/ocular-exposure + animal pharmacodynamic evidence	Timolol solution; Mg(OH)_2_ microparticles + timolol	0.5% timolol; PK AUC to 90 min; repeated NP exposure 14 d for safety	Flux 8.3 ± 2.1 → 12.0 ± 1.1 nmol·cm^−2^·min^−1^; aqueous-humor AUC0–90 min 906 ± 168 → 2845 ± 197 μM·min	Significantly greater IOP lowering than comparator conditions	No observable corneal toxicity after 14 d	NP acts as permeability modifier within a combined topical system, rather than as the drug reservoir	Mechanism incompletely resolved; duration and human relevance require further study

The table compares complete delivery systems rather than nanoparticle chemistry alone. ‘Effect attribution’ indicates whether the reported pharmacokinetic, transport, or pharmacodynamic performance can reasonably be attributed predominantly to the nanoscale formulation or whether it reflects a combined contribution from the nanoparticle, external vehicle, administration route, device, or physical delivery method. Quantitative outcomes retain the definitions, doses, comparator conditions, and time windows of the respective source studies and should not be interpreted as standardized effect sizes across rows. Similarly, reported pharmacodynamic durations should be interpreted within the complete route-specific delivery strategy and not as directly comparable measures of superiority across topical, periocular, intracameral, intravitreal, contact-lens, or externally assisted systems. Quantitative ratios and percentage changes retain the definitions and comparator conditions of the source studies and should not be interpreted as standardized effect sizes across formulations.

**Table 2:** Evidence framework for interpretation of nanoengineered glaucoma drug-delivery studies.

Evidence Label	What This Level Can Establish	What This Level Does Not Establish
In vitro/formulation evidence	Particle or formulation characteristics, drug loading, encapsulation, dissolution/release, physicochemical stability, and in vitro cytotoxicity	Ocular-tissue penetration, intact-eye pharmacokinetics, or therapeutic IOP lowering
Ex vivo transport evidence	Corneal or ocular-tissue permeation, retention, mucoadhesion, and transport across isolated ocular tissues	Drug disposition in the intact eye or in vivo pharmacodynamic efficacy
Animal pharmacokinetic/ocular-exposure evidence	Ocular concentration–time profiles, tissue distribution, residence time, and experimental ocular exposure	Human efficacy, clinically relevant dosing interval, or comparative clinical benefit
Animal pharmacodynamic evidence	In vivo IOP response or another predefined glaucoma-relevant pharmacodynamic endpoint in an animal model	Human clinical efficacy, adherence benefit, long-term clinical safety, or disease progression
Clinical trial/human feasibility evidence	Feasibility of administration in humans, preliminary short-term IOP response, and initial tolerability	Adequately established superiority, non-inferiority, equivalence, long-term effectiveness, adherence benefit, or glaucoma-progression benefit
Comparative clinical efficacy	Comparative therapeutic effect when evaluated in an appropriately designed and adequately powered superiority, non-inferiority, or equivalence trial	Long-term effectiveness, chronic safety, adherence benefit, or disease modification unless specifically assessed
Long-term clinical effectiveness	Sustained disease control, chronic safety and tolerability, treatment burden/adherence, and clinically meaningful long-term outcomes	—
Approved/clinically available nanoparticle-specific glaucoma system	Regulatory approval and routine clinical availability	None identified in the evidence base reviewed here

The first five labels are subsequently applied to the individual primary studies according to the highest evidentiary domains directly evaluated. No nanoparticle-specific glaucoma study identified in this review reached the levels of adequately powered comparative clinical efficacy, long-term clinical effectiveness, or routine approved clinical availability. Abbreviations: IOP, intraocular pressure. The table is intended to clarify interpretation rather than to provide an exhaustive classification of all included studies.

**Table 3:** Human clinical studies of nanoengineered drug-delivery systems for glaucoma included in this review.

Study	Nanoparticle Type	Drug	Administration Route	Clinical Stage/Design	Population	Comparator	Main Outcome	Safety/Tolerability	Current Development Status
Wong et al. [28]	Liposomal formulation	Latanoprost	Subconjunctival injection	First-in-human, open-label, non-comparative pilot study; formal clinical phase not designated	*n* = 6; 5 with ocular hypertension and 1 with primary open-angle glaucoma	None	Mean IOP decreased from 27.55 ± 3.25 to 14.52 ± 3.31 mmHg at 1 h; a ≥20% IOP reduction remained statistically significant through 3 months	No loss of visual acuity; transient discomfort was common immediately after injection; one subject with pre-existing ocular-surface disease reported discomfort and intermittent blurred vision that improved with lubricants	Early human feasibility only. Comparative efficacy, adequately characterized long-term safety, adherence benefit, and routine clinical availability remain unestablished.
Gudmundsdottir et al. [48]	γ-Cyclodextrin nanoparticles	Dorzolamide 3%	Topical	Prospective randomized, single-masked crossover study; formal clinical phase not designated	*n* = 17 untreated subjects with IOP > 18 mmHg	Commercial dorzolamide (Trusopt) administered three times over 24 h	At 4 h, IOP decreased by 3.8 ± 2.6 mmHg (18%) with nanoparticles versus 3.1 ± 3.7 mmHg (14%) with Trusopt; *p* = 0.97 between treatments. No significant between-treatment difference was observed at 24 h	Burning was lower with nanoparticles (12 ± 15 vs. 37 ± 30; *p* = 0.0038); no significant differences in visual acuity or conjunctival hyperemia	Early human feasibility only. The study does not establish superiority, non-inferiority or equivalence, long-term clinical effectiveness, or routine clinical availability.

No nanoparticle-specific glaucoma formulation identified in this review has progressed beyond early human feasibility to adequately powered comparative clinical efficacy or routine approved clinical availability.

**Table 4:** Structured characteristics of primary studies included in the formulation–performance evidence synthesis.

Ref.; Year	Drug	Carrier Composition/Architecture	Particle Size/PDI	Evidence Level; Route; Model; Sample Size	Comparator	Dose/Regimen; Follow-Up	Main PK/Ocular Exposure	Main PD/IOP Findings	Tolerability/Safety	Key Limitation
[25]; 2009	Timolol	Phospholipid surface-modified SLNs	37.7–47.2 nm; PDI NR	Ex vivo transport evidence; ex vivo bioengineered human cornea; *n* NR	Free timolol; unmodified SLNs	NR	Papp 1.33–2.31 × 10^−5^ vs. 4.27 × 10^−5^ cm/s free drug; sustained permeation	Not assessed	NR	Ex vivo only; no in vivo IOP efficacy
[26]; 2020	Pilocarpine	Hollow PLA nanoparticles with tunable shell thickness	Shell ~10–100 nm; PDI NR	Animal pharmacodynamic evidence; intracameral; experimental glaucoma in rabbits; *n* NR	Different shell architectures	Single dose; >56 d	~40-nm shell: EE 86%; therapeutic levels > 56 d	Ocular-hypertension control > 56 d	Preservation of corneal endothelial, retinal and optic-nerve structure/function	Preclinical; loading–release trade-off
[27]; 2020	Pilocarpine	Chitosan/ZM241385-functionalized hollow ceria nanoparticles	NR	Animal pharmacodynamic evidence; topical; experimental glaucoma; *n* NR	Conventional pilocarpine	Single dose; 7 d	~250-fold greater ciliary-body bioavailability	Activity 7 d vs. ~4 h conventional drops	Antioxidant/anti-inflammatory activity reported	Preclinical; complex multifunctional platform
[28]; 2014	Latanoprost	Liposomal formulation	NR	Clinical trial/human feasibility evidence; subconjunctival; human OHT/POAG; *n* = 6	None	Single injection; 3 mo	NR	27.55 ± 3.25 → 14.52 ± 3.31 mmHg at 1 h; ≥20% reduction significant through 3 mo	No VA loss; transient discomfort; one subject with pre-existing OSD had discomfort/blur	Open-label, non-comparative, non-randomized; *n* = 6
[30]; 2024	Latanoprost	Synthetic phosphatidylcholine liposomes + HA + osmoprotectants	195.14 ± 14.34 nm; PDI 0.15	Animal pharmacodynamic evidence; topical; rabbits + cell models; *n* NR	Marketed latanoprost	Single dose; 48 h	Not directly quantified	Effect 48 h vs. 24 h comparator; IOP-effect AUC-derived relative ocular bioavailability 2.97-fold	Cell viability > 80%; no significant surface damage/discomfort; minor hyperemia	Short-term preclinical evidence
[31]; 2025	Latanoprost	HA-coated preservative-free liposomes	NR	Animal pharmacodynamic evidence; topical/ex vivo; porcine tissues + rabbits; *n* NR	Marketed benchmark; saline for selected outcomes	Daily ×15 d; safety monitoring 25 d	Greater latanoprost-acid penetration	IOP reduction 2.51–3.88 mmHg, days 5–15	Ocular-surface parameters broadly physiological	Efficacy only 15 d; chronic evidence lacking
[32]; 2018	Brimonidine	NLC vs. SLN	151.97 ± 1.98 nm; PDI 0.230 ± 0.01	Animal pharmacodynamic evidence; topical; animal model; *n* NR	SLN; commercial drops	>24 h	NLC permeability coefficient 1.227-fold vs. SLN, NS	NLC max reduction 13.14 ± 1.28 mmHg; both active > 24 h	Histological anterior-segment assessment	Preclinical; greater duration not simply predicted by permeability
[33]; 2019	Brimonidine	Span-60 proniosomal gel-derived niosomes	810.95 ± 16.76 nm; PDI 0.6785 ± 0.213	Animal pharmacodynamic evidence; topical; rabbits; *n* NR	Alphagan P	Single dose; 24 h	Not directly assessed	Max IOP reduction 41.32 ± 2.42%; PD AUC0–24 5.024-fold vs. Alphagan^®^ P; PD-derived MRT 7.90-fold; effect remained at 24 h	No Draize-detectable irritation	Preclinical
[34]; 2018	Brinzolamide	TPGS-modified nanoliposomes	96.87 ± 4.43 nm; PDI 0.188 ± 0.011	Animal pharmacodynamic evidence; topical; preclinical ocular model; *n* NR	AZOPT; unmodified liposomes	Up to 11 h	Papp 10.2-fold vs. AZOPT; corneal concentration 3.18-fold higher at 2 h	Effective IOP reduction 3–11 h vs. 3–6 h AZOPT	No significant corneal injury	Preclinical
[35]; 2018	Timolol + brinzolamide	Dual-drug NLC	110.36 ± 0.47 nm; PDI 0.24 ± 0.00	Ex vivo transport evidence; in vitro/ex vivo; *n* NR	Drug suspensions	24 h	Ex vivo penetration prolonged for both agents	Not assessed	NR	No in vivo pharmacodynamic evaluation
[36]; 2020	Travoprost	Nanoemulsion	157.40 ± 0.36 nm; PDI 0.56 ± 0.00	Animal pharmacodynamic evidence; topical; rabbits; *n* NR	Travatan	Up to 60 h	Cmax 1.205 vs. 0.57 µg/mL; AUC 8.032 vs. 4.17 µg·h/mL	Normal IOP maintained 60 h vs. 36 h	Non-irritant	Preclinical
[37]; 2023	Travoprost	Glyceryl-monooleate liquid-crystalline nanostructures	216.20 ± 6.12/129.40 ± 11.73 nm; PDI 0.27 ± 0.03/0.34 ± 0.03	Animal pharmacodynamic evidence; topical/ex vivo; rabbits; *n* NR	Travatan; drug solution	Up to 72 h	Relative bioavailability 106.1%/322.82%; Cmax and Tmax increased	IOP effect 48/72 h vs. 36 h	No ocular injury detected	Preclinical; performance formulation-specific
[38]; 2020	Travoprost	Self-assembled amphiphilic DNA nanoparticles + aptamer	~10 nm; PDI NR	Animal pharmacodynamic evidence; topical; rats + porcine cornea; IOP 20 vs. 18 eyes	Free travoprost/free aptamer	Single dose; PD at 12 h	~4-fold greater delivery at 4 h	Responders 50% vs. 33%; difference in IOP response NS, *p* = 0.1	NR	Improved exposure did not establish superior IOP efficacy
[39]; 2005	Acetazolamide	Span-40/60 REV and multilamellar niosomes	NR	Animal pharmacodynamic evidence; topical; normotensive rabbits; *n* NR	Free 1% acetazolamide; plain niosomes	Single dose 8 h; repeated ×40 d	Release dependent on surfactant/cholesterol/lamellarity	Optimized systems active through 8 h	Slight reversible stromal edema after 40 d	Normotensive model; highest entrapment ≠ best PD performance
[40]; 2017	Timolol	Chitosan nanoparticles	190.9 nm; PDI NR	In vitro/formulation evidence; in vitro; *n* NA	NR	Release to 12 h	49.11 ± 0.49% release at 12 h	Not assessed	NR	No ex vivo or in vivo evidence
[41]; 2018	Timolol	Gelatin nanoparticles	205.8 ± 1.34 nm; PDI 0.014 ± 0.012	Animal pharmacodynamic evidence; topical; experimentally hypertensive rabbits; *n* NR	Marketed timolol	PD 24 h; histology to 3 wk	Sustained release after initial burst	Mean IOP ~20 vs. ~28 mmHg; greater AUC	No Draize irritation; early edema regressed	Preclinical
[42]; 2026	Timolol	Low-MW chitosan/timolol complexes	185–258 nm; liquid formulations 65–130 nm; PDI NR	Ex vivo transport evidence; ex vivo sheep cornea; *n* NR	NR	Release 24 h	Flux 2.54 ± 0.04 μg·cm^−2^·h^−1^; 2.62-fold permeation increase	Not assessed	NR	No in vivo pharmacodynamics
[43]; 2013	Brimonidine	PCL, PLGA and PLA nanoparticles in different vehicles	117.33–131.67 nm; PDI NR	Animal pharmacodynamic evidence; topical; high- and normal-IOP mice; *n* NR	Alphagan P	NR	NR	Peak effect not greater; delayed Tmax, prolonged effect and increased AUC	NR	Carrier and external vehicle changed simultaneously
[44]; 2015	Brimonidine	Chitosan/alginate bioadhesive nanoparticles	115.67 ± 3.58/157.67 ± 5.53 nm; PDI NR	Animal pharmacodynamic evidence; topical; animal model; *n* NR	Alphagan P	Up to 25.2 h	NR	Tmax 5.6–7.2 vs. 2.2–2.4 h; duration 17.2–25.2 vs. 7.0–7.4 h	Cell viability 88.78–97.43%	Longer duration without greater peak efficacy
[45]; 2020	Dorzolamide	Chitosan-decorated PCL nanoparticles	192.38 ± 6.42 nm; PDI 0.18 ± 0.04	Ex vivo transport evidence; ex vivo ocular tissue; *n* NR	Dorzolamide solution	Release to 12 h	Permeation 195.23 vs. 94.23 μg/cm^2^; mucoadhesion increased	Not assessed	Favorable histopathology/HET-CAM	No in vivo IOP assessment
[46]; 2017	Brinzolamide	PLGA nanoparticles A19/B11	193 ± 0.40/660.75 ± 51.61 nm; PDI NR	Animal pharmacodynamic evidence; subconjunctival; rabbits; *n* NR	Brinzolamide suspension	Single injection; 10 d	Size-dependent prolonged exposure	IOP-decrease AUC0–10 d 9.2-/7.1-fold vs. suspension; effect up to 10 d	Transient bleb/vascularization; mild reversible tissue changes	Incomplete in vitro–in vivo correlation
[47]; 2014	Brinzolamide	HPMC-stabilized drug nanocrystals	460 ± 10/530 ± 2 nm; PDI 0.21 ± 0.17/0.12 ± 0.02	Animal pharmacodynamic evidence; topical; laser-induced OHT rats; *n* NR	Azopt; unmilled drug	Single 100-μg dose; acute follow-up	~100% dissolution within 1 min	Best formulation 71.4 ± 5.0% reduction at 60 min vs. 49.6 ± 16.5% Azopt	Mild/no toxicity after relevant dilution	Acute animal efficacy only
[48]; 2014	Dorzolamide	γ-Cyclodextrin nanoparticles	NR	Clinical trial/human feasibility evidence; topical; humans with IOP > 18 mmHg; *n* = 17	Trusopt	One NP dose vs. 3 Trusopt doses; 24 h	NR	4-h reduction 18% vs. 14%, *p* = 0.97; no difference at 24 h	Burning significantly lower; VA/hyperemia similar	Small, short-term human study
[49]; 2017	Dorzolamide	PLGA/PEG nanofiber-derived mucoadhesive microparticles	1.61 ± 0.17 μm final particles; PDI NR	Animal pharmacodynamic evidence; topical; preclinical; *n* NR	Trusopt; intermediate formulations	Same dose; 10 h	NR	Max decrease 24.40 ± 2.31%; duration 10 h vs. 5 h; IOP-decrease AUC 115.85 ± 12.30 vs. 51.46 ± 7.74	NR	Preclinical; final dosage form microscale
[50]; 2013	Pilocarpine	Eudragit RS100 or Gelucire 44/14 nanoparticles ± octadecylamine	Nanometer range; PDI NR	In vitro/formulation evidence; in vitro/formulation; *n* NA	Alternative formulations	Storage 6 mo	Entrapment 31–85%; stability composition-dependent	Not assessed	NR	No PK/PD efficacy
[51]; 2006	Pilocarpine	Chitosan/Carbopol nanoparticles	294 ± 30 nm loaded; PDI NR	Animal pharmacodynamic evidence; topical; rabbits; *n* NR	Liposomes, gel, eye drops	24 h	Slowest release among compared systems	Miosis prolonged to 24 h; largest miosis AUC	NR	Miosis rather than IOP endpoint
[55]; 2019	Timolol + latanoprost	mPEG–PLA micelles in contact lens	20.96 ± 6.05 nm; PDI 0.068	Animal pharmacodynamic evidence; contact lens; elevated-IOP rabbits; *n* NR	Eye drops	>168 h	MRT +79.6-/122.2-fold; bioavailability +2.2-/7.3-fold	IOP reduction >168 h; pharmacological availability 9.8-fold	NR	Rabbit model; no chronic human wear
[56]; 2019	Timolol	Gold nanoparticles + contact lens	GNPs 65 ± 5 nm; PDI 0.12	Animal pharmacodynamic evidence; contact lens; in vitro/in vivo animal; *n* NR	Conventionally soaked lens	72 h	~4-fold iris–ciliary and ~2-fold conjunctival exposure at 24 h	Both lens systems prolonged IOP reduction 72 h	Lens swelling/transmittance not significantly altered	Greater loading/exposure without major PD advantage
[57]; 2022	Latanoprost	PLGA nanoparticles + iontophoresis	313 ± 43 nm; PDI 0.05	Animal pharmacodynamic evidence; iontophoretic delivery; preclinical; *n* NR	Xalatan	4 mA × 30 min; single treatment; exposure to 12 d	Drug AUC > 23-fold vs. Xalatan	IOP-effect AUC > 16-fold; effect > 7 d	No significant damage on reported assessments	Combined NP–iontophoresis strategy; NP tablet ~170–187 μg latanoprost vs. 0.175 μg Xalatan^®^ drop; AUC ratios not dose-normalized; single-treatment preclinical evidence
[58]; 2020	Brinzolamide + miRNA-124	PEG–PSA nanoparticles	250–400 nm; PDI NR	Animal pharmacodynamic evidence; intravitreal; microbead-OHT and ON-crush models; *n* NR	PBS/untreated	Release to day 12	Sustained release days 1–12	IOP fell 22.50 ± 3.89 → 12.83 ± 1.47 mmHg by day 6; RGC outcomes improved	NR	Experimental intravitreal strategy; IOP lowering and RGC outcomes assessed in separate disease models; does not establish disease-modifying efficacy in glaucoma
[59]; 2017	Timolol + Mg(OH)_2_	Mg(OH)_2_ nanoparticles as permeability modifier	71 nm; PDI NR	Animal pharmacodynamic evidence; topical co-instillation; experimental ocular models; *n* NR	Timolol alone; microparticles	AUC to 90 min; NP exposure 14 d	Flux 8.3 ± 2.1 → 12.0 ± 1.1 nmol·cm^−2^·min^−1^; aqueous-humor AUC0–90 min 906 ± 168 → 2845 ± 197 μM·min	Significantly greater IOP lowering	No observable corneal toxicity after 14 d	Mechanism of permeability enhancement unresolved

Quantitative PK and PD measures retain the endpoint definitions, units, denominators, comparator conditions, and time windows of the respective primary studies and are not directly comparable across rows. NR, not reported in the information extracted for the present review; AUC, area under the curve; EE, encapsulation efficiency; HA, hyaluronic acid; IOP, intraocular pressure; MRT, mean residence time; NLC, nanostructured lipid carrier; OHT, ocular hypertension; PCL, polycaprolactone; PDI, polydispersity index; PLA, poly(lactic acid); PLGA, poly(lactic-co-glycolic acid); SLN, solid lipid nanoparticle; VA, visual acuity. Where a requested study characteristic was not reported in the information extracted for the present review, the corresponding field is designated as NR rather than inferred from other information.

**Table 6:** Route-specific comparison of nanoengineered glaucoma delivery strategies according to ocular barriers, invasiveness, representative glaucoma efficacy/residence, and safety considerations. Reported outcomes and durations are study-specific and should not be interpreted as direct efficacy rankings across administration routes.

Route	Main Barriers/Disposition	Invasiveness	Representative Glaucoma Efficacy/Residence	Main Safety Considerations
Topical	Tear clearance, conjunctival loss, corneal epithelial barrier	Non-invasive	Effects ranging from hours to several days depending on system [30,37,47]	Ocular-surface exposure, epithelial toxicity, repeated-dose tolerability
Subconjunctival	Bypasses precorneal clearance; creates local periocular depot; scleral transport remains relevant	Injection	~10 d with PLGA brinzolamide [46]; human liposomal LAT activity through 3 mo [28]	Injection discomfort, bleb, vascularization, depot persistence
Other periocular	Bypasses ocular surface; transscleral transport and choroidal clearance remain relevant	Injection/procedure	Evidence less developed than for subconjunctival delivery	Local tissue reaction, clearance, repeated administration
Intracameral	Direct anterior-chamber placement	Invasive intraocular	>56 d experimental IOP control with hollow PLA NPs [26]	Corneal endothelium, inflammation, persistence, retreatment
Intravitreal	Bypasses anterior barriers; vitreous distribution and retinal access dominate	Invasive intraocular	Experimental RGC-directed/molecular delivery [58]	Inflammation, retinal toxicity, carrier persistence, procedural risk
Suprachoroidal	Direct access to suprachoroidal compartment; avoids corneal/anterior-chamber entry barriers	Minimally invasive intraocular/periocular injection	Liposomal latanoprost lowered rabbit IOP for up to 56 d [71]	Distribution within suprachoroidal space, choroidal/retinal exposure, procedural safety, repeatability

## Data Availability

No new data were created or analyzed in this study. Data sharing is not applicable to this article.
